# Pattern-recognition receptors in endometriosis: A narrative review

**DOI:** 10.3389/fimmu.2023.1161606

**Published:** 2023-03-23

**Authors:** Bao Guo, Jia hua Chen, Jun hui Zhang, Yuan Fang, Xiao jing Liu, Jing Zhang, Hai qing Zhu, Lei Zhan

**Affiliations:** ^1^ Department of Obstetrics and Gynecology, The Second Affiliated Hospital of Anhui Medical University, Hefei, Anhui, China; ^2^ First Affiliated Hospital of Anhui Medical University, Hefei, Anhui, China; ^3^ Second Affiliated Hospital of Anhui Medical University, Hefei, Anhui, China

**Keywords:** endometriosis, pattern-recognition receptors, TLRs, CLRs, NLRs, RLRs, ALRs, immune response

## Abstract

Endometriosis is closely associated with ectopic focal inflammation and immunosuppressive microenvironment. Multiple types of pattern recognition receptors (PRRs) are present in the innate immune system, which are able to detect pathogen-associated molecular patterns (PAMPs) and danger-associated molecular patterns (DAMPs) in both intracellular and external environments. However, the exact role of PRRs in endometriosis and the underlying molecular mechanism are unclear. PRRs are necessary for the innate immune system to identify and destroy invasive foreign infectious agents. Mammals mainly have two types of microbial recognition systems. The first one consists of the membrane-bound receptors, such as toll-like receptors (TLRs), which recognize extracellular microorganisms and activate intracellular signals to stimulate immune responses. The second one consists of the intracellular PRRs, including nod-like receptors (NLRs) and antiviral proteins retinoic acid-inducible gene I (RIG-I) and melanoma differentiation-associated gene 5 (MDA-5) with helix enzyme domain. In this review, we mainly focus on the key role of PRRs in the pathological processes associated with endometriosis. PRRs recognize PAMPs and can distinguish pathogenic microorganisms from self, triggering receptor ligand reaction followed by the stimulation of host immune response. Activated immune response promotes the transmission of microbial infection signals to the cells. As endometriosis is characterized by dysregulated inflammation and immune response, PRRs may potentially be involved in the activation of endometriosis-associated inflammation and immune disorders. Toll-like receptor 2 (TLR2), toll-like receptor 3 (TLR3), toll-like receptor 4 (TLR4), nod-like receptor family caspase activation and recruitment domain (CARD) domain containing 5 (NLRC5), nod-like receptor family pyrin domain containing 3 (NLRP3), and c-type lectin receptors (CLRs) play essential roles in endometriosis development by regulating immune and inflammatory responses. Absent in melanoma 2 (AIM2)-like receptors (ALRs) and retinoic acid-inducible gene I-like receptors (RLRs) may be involved in the activation of endometriosis-associated immune and inflammation disorders. PRRs, especially TLRs, may serve as potential therapeutic targets for alleviating pain in endometriosis patients. PRRs and their ligands interact with the innate immune system to enhance inflammation in the stromal cells during endometriosis. Thus, targeting PRRs and their new synthetic ligands may provide new therapeutic options for treating endometriosis.

## Introduction

Endometriosis is a gynecological disorder characterized by the presence of endometrial glands and stroma with growth function outside the uterine cavity, which often causes clinical symptoms such as chronic pelvic pain, infertility, and even tumors (1). Increased local production of large amounts of progesterone by endometriotic stromal cells combined with progesterone receptors deficiency lead to progesterone resistance, which is associated with their reduced capacity for decidualization (2). Moreover, aberrant activity of aromatase enzyme combined with 17β-hydroxysteroid dehydrogenase type 2 deficiency as a consequence of progesterone resistance contribute to the abnormally high levels of estradiol in the endometriotic tissue of endometriosis patients (3, 4). Endometriotic stromal cells also secrete large amounts of immune mediators such as interleukin-1β (IL-1β), IL-6, tumor necrosis factor-α (TNF-α), RANTES (regulated upon activation, normal T cell expressed and secreted), and MCP-1 (monocyte chemoattractant protein-1) (5). Proliferation and invasiveness of endometriotic stromal cells are supported by different chemokines produced in the endometriotic microenvironment such as RANTES (CCL5), CCL2, CXCL8, and thymus-expressed chemokine (CCL25) (6). It is suggested that argument induced by inflammatory mediators such as IL-1β may be through extracellular regulated protein kinases (ERK) signal pathway (7). Epidemiological survey shows that the incidence of endometriosis in women of childbearing age is about 10%, in infertile women it can be as high as 25%-50%, and the risk of cancer in patients with endometriosis is also significantly increased (8). Long-term psychological stress seriously affects the quality of life of women in their reproductive age and causes heavy economic burden to their families and the society at large (9). However, little is known about the mechanisms associated with the occurrence and development of endometriosis (10), which poses a major challenge for the development of efficacious therapeutic agents for treating endometriosis, causing the average annual recurrence rate of endometriosis after treatment to reach over 10% (11). Therefore, it is urgent to alleviate the clinical symptoms related to endometriosis and reduce the recurrence rate after treatment in the clinic. Understanding the mechanisms underlying the pathophysiology of endometriosis is paramount to improve the therapeutic efficacy of existing clinical therapeutic options for endometriosis (12).

As early as 1927, Sampson proposed that the occurrence and development of endometriosis was closely related to blood reflux. This mechanism proposes that during menstrual period in women, endometrial cells and tissue fragments survive through tubal reflux, adhere to and invade the pelvic structure, leading to ectopic lesions through the three processes of “adhesion-invasion-vascularization”. Data indicates that patients with carbohydrate antigen 125 (CA125) ≥ 35 U/mL show a higher risk for pelvic adhesions. Moreover, a previously published study identified important proangiogenic factor such as vascular endothelial growth factor (VEGF), IL-1β, IL-6 and IL-8 as well as TNF-α played important roles in the vascularization process in endometriosis (13, 14). This mechanism is also recognized as the classical mechanism of endometriosis (15). However, Sampson’s blood reflux theory cannot fully explain the difference between the incidence rate of blood reflex (90%) and the incidence rate of endometriosis (10%) (16). In recent years, based on the clinical symptoms presented by endometriosis patients, studies (17, 18) have proposed inflammation and immune mechanisms to be closely related to endometriosis. Firstly, the main reason for infertility in endometriosis patients is the formation of an endometrial microenvironment that is not conducive to embryo implantation, including the formation of anatomical scars and the adverse effects of local inflammation on oocyte quality and early embryonic development. Studies have shown that many important inflammatory mediators are significantly elevated in the ectopic lesions of endometriosis patients (19, 20). The formation of chronic inflammatory microenvironment in the endometrium affects the quality and maturation of oocytes (21). Low-quality embryos have lower ability to implant, which is not conducive to pregnancy (22). Secondly, studies have shown that the inflammatory microenvironment of ectopic lesions in endometriosis patients activates sensory nerve endings, and further increases the secretion of inflammatory mediators, resulting in the transmission of pain stimulation to the spinal cord, causing and maintaining chronic pain in these patents (23, 24). Thirdly, endometrial microenvironment immune disorder in endometriosis patients is also one of the important reasons for infertility and pelvic pain (25). Studies have shown that, based on Sampson’s theory of countercurrent blood flow, the impaired immune surveillance of autologous cells and the overload on the immune system to remove the endometrial debris promotes immune disorder, which leads to the dysregulation of of innate and acquired immune cell groups (such as CD8^+^ T cells, CD56^+^ NK cells, CD163^+^ macrophages and so on) in endometriosis patients (26, 27). In view of the above mechanisms, researchers also consider endometriosis as an inflammation and immune-related disease, and identifying molecules that specifically target such inflammatory and immune response mechanisms in the endometrium will be the key to improving the quality of life of endometriosis patients (28).

Pattern recognition receptors (PRRs) (**Table 1**) recognize pathogen-associated molecular patterns (PAMPs) and can distinguish pathogenic microorganisms from self (35–40). PAMPs are highly conserved structures in various microorganisms (41), including bacterial lipopolysaccharide, lipoprotein, peptidase, flagellin, non-methylated CpG dinucleotide motifs (CpG-DNA), viral double-stranded RNA, fungal cell wall and so on (42). PRRs trigger receptor ligand reaction after recognizing PAMPs, followed by the transmission of microbial infection signals to the cells to stimulate the host immune response, thereby removing the pathogenic microorganisms (43). On the basis of protein domain homology, PRRs can be divided into five groups (44), namely, the toll-like receptors (TLRs), c-type lectin receptors (CLRs), nod-like receptors (NLRs), retinoic acid-inducible gene I-like receptors (RLRs), and absent in melanoma 2 (AIM2)-like receptors (ALRs). The two major classes of these families are membrane-bound receptors and unbound intracellular receptors (45). The first class consists of TLRs, which are mainly membrane bound receptors but may also be localized in intracellular compartment occasionally (e.g., TLR3) and CLRs, which are located in endocytic compartments or at the cell surface (46, 47). These receptors identify microbial ligands in endosomes and the extracellular environment. The second category consists of the NLRs, RLRs, and ALRs and they are mainly found in the cytoplasm and recognize intracellular pathogens in the cytoplasm (35). The generation of pro-inflammatory cytokines and interferons (IFN), which are essential for triggering both innate and adaptive immune responses, is a fundamental aspect of a PRRs-induced innate immune response (48). Additionally, the activation of PRRs triggers non-transcriptional reactions, which includes the induction of phagocytosis, autophagy, cell death, and cytokine processing (49). Through carefully regulated signal transduction pathways, these transcriptional and non-transcriptional innate immune responses are linked to PRRs-mediated microbial recognition (50). Immune responses are orchestrated by the synchronization of various signaling pathways, which prevent the spread of an initial infection and guide the proper adaptive response (51). Endometriosis may develop in at least two waves. Bacterial infection, for example with *Escherichia coli*, is the first stage. The second wave of chronic and aseptic inflammation leading to tissue damage may be caused due to a combination of apoptosis inhibition and persistent inflammation, redox-active iron-dependent oxidative stress, activation of PAMPs/danger-associated molecular patterns (DAMPs) receptors and DAMPs released by the damaged cells. In conclusion, endometriosis development is intimately linked to the original infection and subsequent aseptic inflammation (52). However, it is still unclear how PRRs and endometriosis are related. Conventional medicine has only provided symptomatic pain relief so far. In the current review, we assessed existing literature and examined the association between PRRs and endometriosis, with the hope to improve our understanding of the role of PRRs in endometriosis, guide future research, and identify innovative therapeutic strategies to treat endometriosis.

**Table 1:** Pattern-recognition receptor families.

Family	Members	Shared domains	Receptor locations	Inflammatory cytokines	References
TLR	1-10 in humans;1-9, 11-13 in mice	LRR, TIR	Cell surface, endosomal compartments	IL-1β, IL-1, IL-12, IL-18, IL-6, IFN-γ and TNF-α	(25, 29)
CLR	Dectin-1, Dectin-2,... etc.	C-type lectin	Cell surface	IL-6, IL-10, IL-12, IL-17, IFN-γ and TNF-α,	(26, 30)
NLR	NODI (NLRC1), NOD2 (NLRC2), NLRC3-5, NLRP1-9, 11-14;NAIPl, -2,-5, -6	Nucleotide binding, LRR	Cytoplasm, plasma, and cndosomal membrane associated	IL-1β, IL-1 and IL-18	(27, 31)
RLR	RIG-I, MDA5, LGP2	DExD/H helicase	Cytoplasm	IFN	(32, 33)
ALR	AIM2, IFI16	PYRIN, HIN-200	Cytoplasm, nucleus (IFI16)	IL-1β, IL-6, IL-17, IL-8, IL-18, IL-22, CCL2, CCL5, CCL20, IFN-γ and TNF-α	(28, 34)

AIM, absent in melanoma; ALR, AIM2-like receptor; CLR, C-type lectin receptor; IFI, interferon, γ-inducible; LGP, laboratory of genetics and physiology; LRR, leucine-rich repeat; MDA, melanoma differentiation gene; NAIP, NLR family, apoptosis inhibitory protein; NLR, nucleotide-binding oligomerization domain receptor; NLRC, NLR family CARD domain containing; NLRP, NLR family PYD domain containing; NOD, nucleotide-binding oligomerization domain; RIG-I, retinoic acid–inducible gene I; RLR, RIG-I-like receptor; TIR, Toll/IL-1 receptor/ resistance; TLR, Toll-like receptor.

## TLRs and endometriosis

### TLRs

TLRs are type I transmembrane proteins consisting of two domains, namely, a transmembrane domain, which is necessary for downstream signal transmission, and an intracellular domain containing leucine-rich repeats (LRRs), which mediates the recognition of PAMPs or DAMPs (53). The activation of TLRs starts a chain of events that eventually results in the transcription of several downstream genes involved in inflammatory response and antimicrobial defense. There are currently 13 TLRs in the mammalian family. Humans and rats share the same TLR1–9 receptors. In rodents, TLR10 is not functional, and humans do not express TLR11–13 (54). TLRs differ from one another in terms of their ligand specificities, expression patterns, and the downstream signaling pathways that are being activated. TLRs are crucial for controlling inflammation in both infectious and non-infectious disorders (55). The levels of TNF-α, IL-1, IL-6, IL-1β, and transforming growth factor β (TGF-β) in the TLR cascade are known to be altered in endometriosis patients. The levels of IL-1β, one of the pro-inflammatory cytokines secreted upon TLR activation, are significantly higher in the extrauterine tissues and peritoneal fluid of endometriosis patients than that of healthy women (56–59). TLRs activation by PAMPs and/or DAMPs may cause structural and functional alterations that accelerate the development of endometriosis (52).

### TLR4 in endometriosis

**Figure 1:**
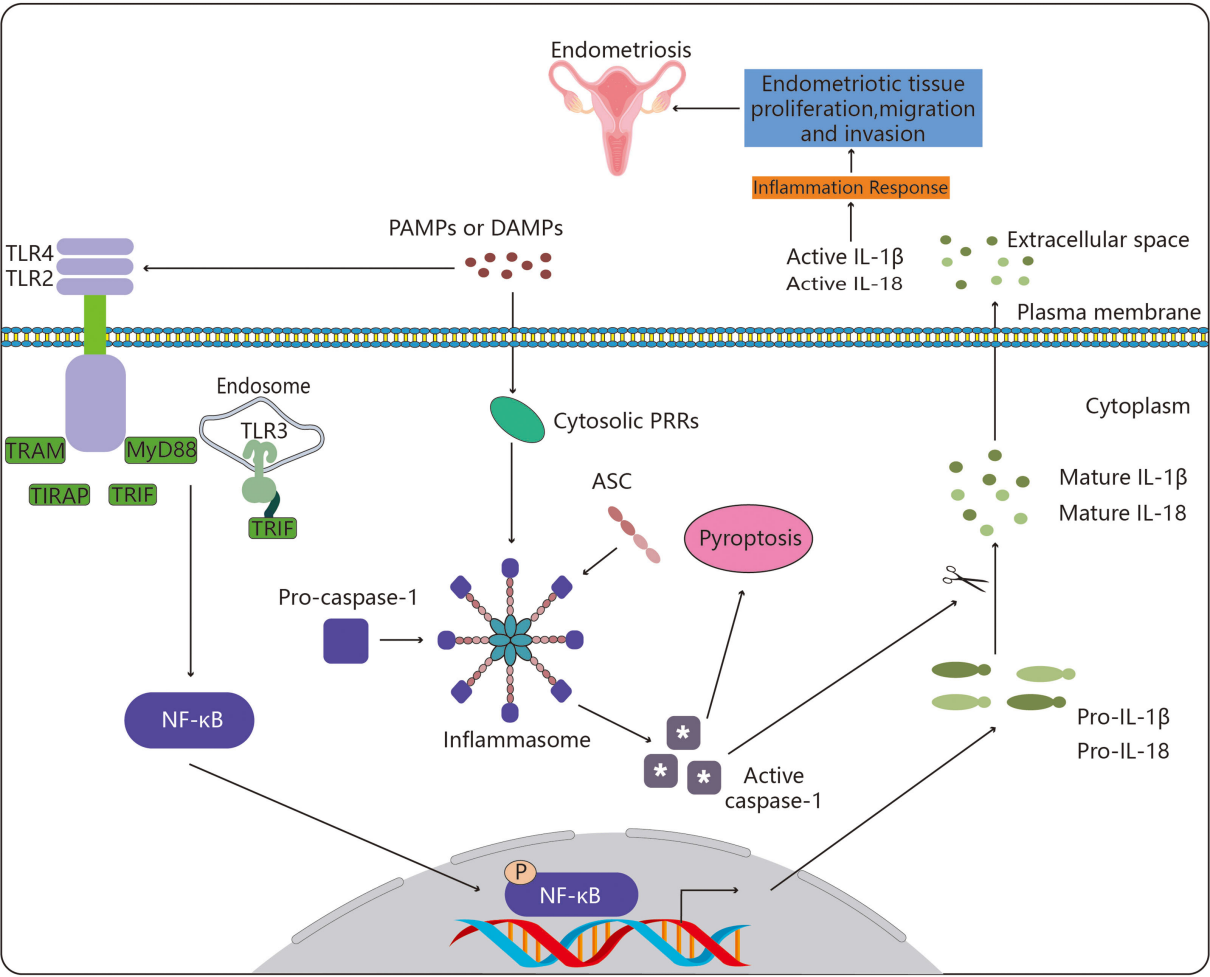
Toll-like receptors in endometriosis. By enlisting particular adaptor molecules such as MyD88, TIRAP, TRAM, and TRIF, TLR4, TLR2 and TLR3 are able to recognize these chemical signals. This sets off a chain of biological processes that activates the transcription factor NF-κB. The pro-inflammatory cytokines IL-1 (pro-IL-1β) and IL-18 are then transcriptionally induced as a result of activated NF-κB translocating to the nucleus. Parallel to this, cytosolic PRRs (NLRs, ALR and Pyrin) also identify DAMPs and PAMPs, and they do so by recruiting the adaptor ASC to create an inflammasome, the assembly of which triggers the cysteine protease caspase-1. The pro-inflammatory form of programmed cell death known as pyroptosis is brought on by caspase-1 activation, which also encourages the proteolytic cleavage and maturation of pro-IL-1β and pro-IL-18. Endometriotic tissue proliferation, migration, and invasion results from the activation of NF-κB-induced proinflammatory cytokines and pyroptosis, ultimately resulting in the development of endometriosis. The meaning of symbol "*" is active caspase-1.

TLR4 is one of the members of the TLRs family. As an acute receptor, TLR4 is widely expressed on the cell membrane of immune cells. Previous studies (60, 61) have shown that TLR4 is expressed by macrophages, dendritic cells, neutrophils, natural killer cells and other immune cells (**Figure 1**). It plays an important role in the onset and development of many diseases and has emerged as a research hotspot in recent years (62, 63). Several studies have validated TLR4 expression in the endometrial cells (64, 65). In 2015, endometrial tissues from 15 patients undergoing laparoscopic surgery were analyzed and the expression of TLR4 protein was confirmed to be higher in the endometrial stromal cells than in the endometrial epithelial cells, suggesting the potential involvement of TLR4 in the regulation of immune response in endometriosis (66). In the same year, another study reported that the expression of TLR4 and TLR2 were higher in the endometrial tissues from endometriosis patients than that of normal endometrium, suggesting the potential role of TLR2 and TLR4 in the development of endometriosis (67). In animal experiments, investigators injected lipopolysaccharide (LPS) into the peritoneum of mouse model of endometriosis and found that the activation of LPS/TLR4 pathway induced the expression of nuclear factor-κB (NF-κB) and promoted its translocation to the nucleus, causing peritoneal macrophages and ectopic endometrial cells to release inflammatory factors (68). LPS was also shown to promote the proliferation and invasion of ectopic endometrial stromal cells by inducing the upregulation of cyclooxygenase-2 (COX-2) and prostaglandin E2 (PGE2). Endometriosis has been associated with an altered profile of intestinal microflora in rhesus monkeys, and it is linked to higher concentrations of gram-negative bacteria. Based on these studies, we speculated that as carriers of LPS, different gram-negative bacteria, such as *Escherichia coli*, residing in the vagina could be involved in the pathogenesis of endometriosis in humans. LPS, one of the most pro-inflammatory mediators, induces the expression of COX-2 in the periphery. Endometriotic lesions have high COX-2 and PGE2 biosynthesis compared with the normal endometrium. The addition of NF-κB inhibitor has been reported to inhibit the effect of LPS on endometriotic lesions in mice (69). In addition, inflammatory cytokines such as TNF-α and IL-6 activate c-jun terminal kinases (JNK) in the endometrial cells of endometriosis patients (70). Activation of the JNK signaling pathway further up-regulates the expression of inflammatory cytokines and promotes endometrial cell proliferation by regulating protein translocation (such as MEKK1/4, MLK1-4, ASK1 and MKK4/7) across the cytoplasm and nucleus (71). Although clinical evidence is still lacking, it can still be boldly speculated from the existing cell and animal experiments that the PAMPs (such as LPS) released by pathogenic microorganisms, which enter the upper reproductive tract with countercurrent blood are recognized by TLR4 on endometrial cells. This activates the TLR4 inflammatory signal pathway and promotes the recruitment and activation of immune cells (such as macrophages), triggers local inflammatory response, and promotes the secretion of different inflammatory factors and growth factors, stimulates the proliferation of endometrial cells, and continuously activates and maintains the inflammatory microenvironment. Also, LPS or PAMPs may furtehr promote the adhesion, invasion or proliferation of endometrial cells, expanding the transmission of the local inflammatory response, and finally leading to the initiation and development of endometriosis. Continuous inflammatory injury also causes the damaged cells to release DAMPs such as heat shock protein 70 (HSP70), high mobility group box-1 (HMGB-1) protein and so on, leading to the activation of TLR4 mediated inflammatory response, and causing further tissue damage. Therefore, such a vicious cycle continues to occur in ectopic endometrial lesions.

### TLR3 in endometriosis

TLR3 is one of the important members of the PRR family, which is essential for innate immunity. By recognizing endogenous and exogenous ligands, it participates in a variety of activities, including cell proliferation, apoptosis, angiogenesis, tissue remodeling, and repair (72). TLR3 signaling (**Figure 1**) also depends on the protein TIR-domain-containing adapter-inducing interferon-β (TRIF). Given the function of TRIF, the TLR3 signaling pathway is focused on the following two directions. NF-κB and the activator protein-1 (AP-1) transcription factor are activated by one pathway, while interferon regulatory factor 3 (IRF3) and IRF7 are activated by the alternate pathway (73). Transforming growth factor-b-activated kinase 1 (TAK1) is activated by TRIF through tumor necrosis factor receptor-associated factors 6 (TRAF6) and a number of other adaptor proteins (**Table 2**). Kinases and mitogen-activated protein kinase (MAPK) are activated by TAK1. By phosphorylating and degrading its inhibitor (i.e. inhibitors of kappa B (IκB)), inhibitory kappa B kinases (IKKs) activate NF-κB. IκB and NF-κB are then transported to the nucleus, where they promote the transcription of genes encoding inflammatory cytokines such as IL-6 and IL-8 (77, 78). TRIF induces interferon expression *via* TRAF3 and subsequently, it enhances type I and type III IFN production and also induces the transcription of a series of IFN-responsive genes. TLR3 is expressed by the endometrial tissue, dendritic cells, macrophages, and fibroblasts, as well as endothelial and epithelial cells.

**Table 2:** Adaptor proteins.

Adaptor or adaptor set	Receptor interaction	Signaling interaction	Localization	References
TIRAP/MyD88	TIR domain	Death domain	Cell surface, endosomal compartments	(74)
TRAM/TRIF	TIR domain	TRAF binding, RHIM domain	Cell surface, endosomal compartments	(55)
MAVS	CARD domain	Proline-rich region, TRAF binding	Mitochondrial, peroxisomal, and mitochondria-associated membranes	(75)
ASC	PYRIN	CARD domain	Cytosol, mitochondria	(76)

ASC, apoptosis-associated speck-like protein containing a CARD; CARD, caspase recruitment domain; MAVS, mitochondrial antiviral signaling protein; RHIM, RIP homotypic interaction motif; TIR, Toll/IL-1 receptor/resistance; TIRAP, TIR-containing adaptor protein; TRAF, TNF receptor–associated factor; TRAM, TRIF-related adaptor molecule; TRIF, TIR domain–containing adaptor-inducing IFN-β.

Moreover, endometrial epithelial tissue expresses TLR3 in a menstrual cycle-dependent manner (79). By simultaneously measuring all TLRs, the above study measured their altered expression profiles, and showed their positive correlation with the expression of the IL-6 and IL-8 genes, suggesting their potential contribution to the inflammatory etiology of adenomyosis (80). Compared to the control endometrium, both the ectopic and the eutopic endometrium showed higher expression levels of TLR3 mRNA and protein (80). They tested the mRNA expression levels of a few selected genes involved in specific signaling pathways (TICAM, NF-κB1A, CXCL10, IRF3, IFN-B1, IL-6, and IL-8) in clinical specimens, to ascertain whether or not the elevated expression of TLR3 gene transcript in the endometriosis tissues resulted in a shift in the expression of downstream signaling molecules (81). TRIF is activated upon TLR3 binding and triggers the activation of NF-κB signaling pathway, which subsequently promotes the release of pro-inflammatory and inflammatory cytokines such as IL-6 and IL-8. Endometriosis patients also showed significant and obvious alterations in the mRNA expression levels of other genes in the TLR3 cascade, indicating that eutopic endometrium experiences an intense inflammatory state similar to that of the ectopic endometrium. These changes were in addition to the elevation in the levels of IL-6 and IL-8. Intriguingly, the findings from the above studies revealed a considerable difference in the expression of the aforementioned genes between ectopic and eutopic endometrium, indicating that the latter is capable of evading immune surveillance due to fundamental changes in immune response. The proliferation and viability of endometrial cells are thought to be enhanced by increased TLR3 expression and consequent increase in the levels of interferons and pro-inflammatory cytokines (82).

### TLR2 in endometriosis

By establishing functional heteromeric complexes with TLR1, TLR4, and TLR6, TLR2 is known to have anti-microbial functions and is involved with lipopeptides that identify bacteria in synergy with these receptors (74, 83). TLR2 (**Figure 1**) may play a crucial role in the pathogenesis of endometriosis patients. In addition to the vast set of TLRs, TLR2 in particular, plays important roles in bacterial, viral, and fungal infections (84), along with immunological roles as a crucial signaling molecule of the innate immune system (85). Researchers assessed the levels of numerous immunological cell subsets (dendritic cells, monocytes, and basic peripheral blood lymphocytes) expressing TLR2 and evaluated the potential correlation between the expression of TLR2 and the clinical characteristics of endometriosis patients (86).

Overall, there are only few reports that have explored TLRs in patients with endometriosis, leaving only a small amount of information related to the presence of TLRs 1, 2, 3, 4, 5 and 6 in the epithelium of the female genital tract at different sites (87). TLR1-6 was reported to be expressed in endometrial samples while TLR10 was not (88). The greatest hurdles on the path to an early diagnosis of endometriosis includes the absence of biomarkers that could be non-invasively utilized to detect endometriosis. Further studies investigating the cost effectiveness of utilizing blood cells expressing TLRs as clinical markers of endometriosis needs to be explored for early disease detection.

## CLRs and endometriosis

### CLRs

The crucial pathogen pattern-recognition molecules known as CLRs are known to identify the shapes of carbohydrates (89). When a ligand binds to a CLR, many cellular responses are brought about, including a respiratory burst, the secretion of cytokines and chemokines, and ultimately the initiation of adaptive immunological responses (90). In recent cutting-edge research, CLRs have been shown to play a crucial role in initiating anti-inflammatory immune responses and maintaining homeostasis in host immunological response (91).

### CLRs in endometriosis

**Figure 2:**
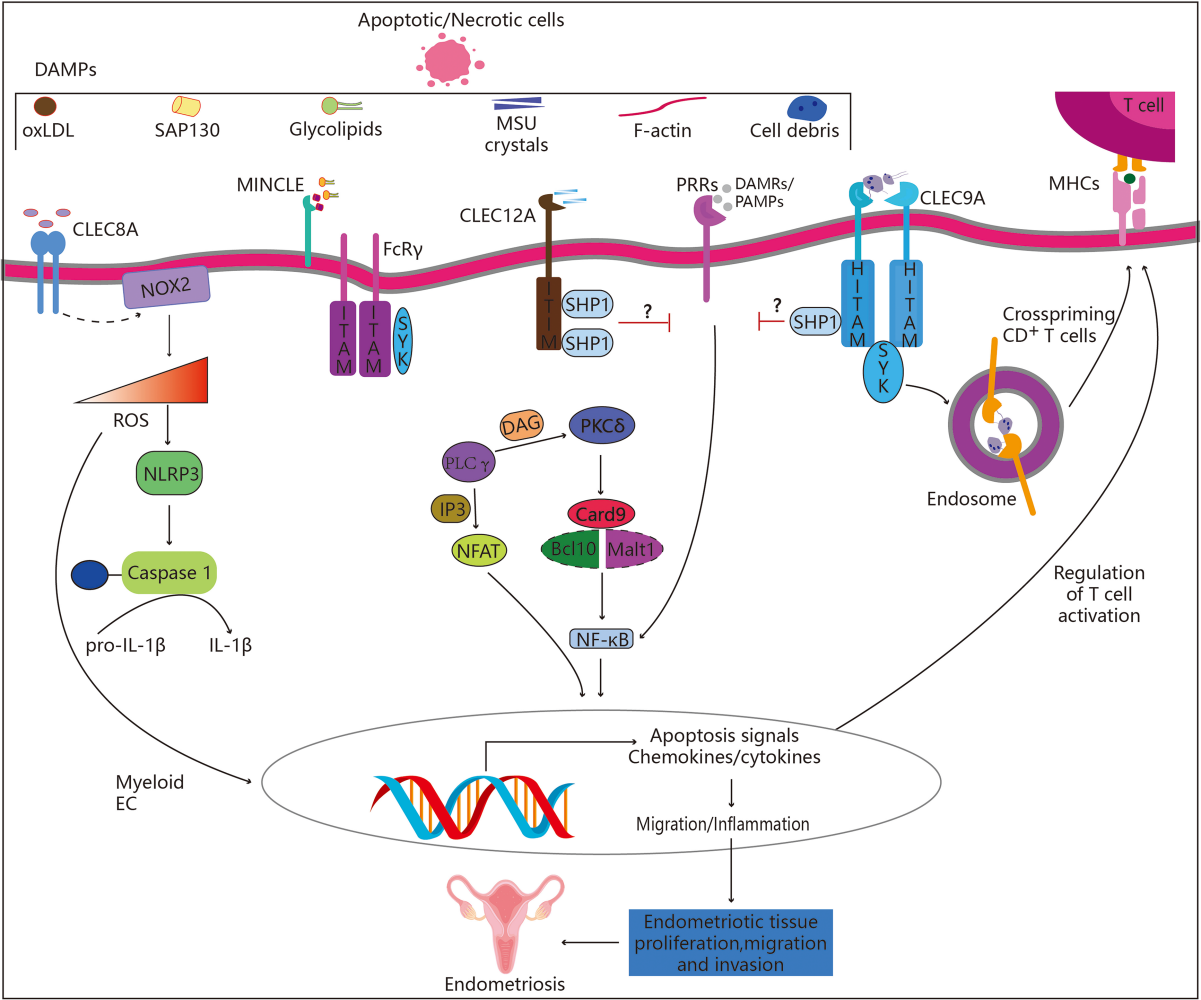
Role of C-type lectin-like receptors in endometriosis. CLRs belonging to the “Dectin family” recognize a variety of self-derived ligands, including pathogen-associated molecular patterns (PAMPs). Binding to PAMPs results in the activation of the immune-receptor tyrosine-based activation motif (ITAM), which attracts and activates kinases from the SYK family. Endometriosis may be aided by the subsequent activation of the Card9-Bcl10-Malt1 complex through SYK, which results in NF-κB activation and the transcription of a number of chemokines and cytokines (CLEC4E alias INCLE). Alternately, increased synthesis of ROS and IL-1 (CLEC8A), which alters gene expression and releases ROS into the extracellular matrix, may control immune response. On the other hand, activation of the immune receptor’s tyrosine-based inhibition motif (ITIM) results in the recruitment and activation of SHP-1 and SHP-2 as well as the dephosphorylation of activation motifs, preventing further activation by other PRRs such as CLEC12A. Hemi-ITAM (HITAM), a component of CLEC9A, is essential for CD8^+^ T cell cross-priming.

Immunoglobulins and CLRs work in synergy and are intimately connected in the etiology of endometriosis (92). Among patients suffering from benign disease, endometriosis and malignancy, a previous study compared the expression levels of mRNAs which encoded CLRs and their adaptive molecules associated with the innate immune reaction, along with their protein expression (31). They found that the etiology of endometriosis was directly related to the combined action of CLRs, its adaptor mRNA molecules, immunoglobulin G (IgG), IgA, and IgM. These CLRs and the associated endometriosis mechanisms are illustrated by a few examples in **Figure 2**. Another study investigated the potential role of mannose receptor, C-type 2 (MRC2) in the development of Treg cells by co-culturing ESCs with mannose receptor C, macrophages and naïve CD4^+^ T cells and along with the knockdown of MRC2 (93). Compared with the vector group, the proportion of Treg cells, in particular CD4 high regulatory T cells (Tregs), was increased in the MRC2-silenced group, which indicated that MRC2 was essential for the emergence of Treg cells within the endometriotic tissue. The peritoneal DCs within the endometriotic tissue exhibited high levels of mannose receptor (MR), making them more capable of phagocytosing dead endometrial stromal cells and enabling the development of endometriosis (94). Endometriosis treatment options could include the modulation of MR expression or activity in peritoneal DCs. The function of DC-SIGN^+^ macrophages within the immunological milieu of endometriosis has been investigated before (95). The number of macrophages within the abdominal immune microenvironment in patients with DC-SIGN^+^ CD169^+^ endometriosis was found to be elevated. When Colony stimulating factor-1 (CSF-1) was added to induce the polarization of macrophages to DC-SIGN^+^ CD169^+^ phenotype and generate DC-SIGN^+^ macrophages, the level of peripheral blood lymphocytes decreased, which was comprised of a high percentage of Treg cells as well as a low percentage of CD8^+^ T cells. Further investigation of the mechanism and biological functions of DC-SIGN^+^ macrophages that are activated by CSF-1 would enable a better understanding of the pathophysiology of endometriosis. There are 4 new somatic mutations in caspase activation and recruitment domain (CARD)10 and CARD11 in 4.0 percent (4/101) of patients with ovarian endometriosis (96). According to the above findings, these mutations were mutually exclusive and promoted a beneficial effect on the pathogenesis of ovarian endometriosis. SRC signaling was active in both the eutopic endometrium of endometriosis patients and in *in vitro* models of endometriosis, and their findings suggested the novel therapeutic potential of Src inhibition (Src-pY416) for treating endometriosis-associated ovarian cancers (EAOCs) (97). Endometriosis exhibited sustained activation and dysregulation in the T-cell immunoglobulin and mucin domain-3 (TIM-3)/Galectin-9-dependent pathway, which likely induced a weakening of immune surveillance mechanisms, promoting the survival of ectopic lesions, eventually contributing to the progression of reproductive failure in endometriosis patients (98). Serum soluble triggering receptor expressed on myeloid cells-1 (sTREM-1) may serve as a prognostic indicator for female fecundity, possibly due to poor immune system inflammation (99). By comparing follicular fluid, serum, and endometriosis loci with the peritoneal fluid, they suggest that the function of sTREM-1 needs to be re-examined in endometriosis patients.

## NLRs and endometriosis

### NLRs

NLRs are the regulatory nexuses for a variety of biological processes and are a family of widely used and highly developed signaling regulators (100). These proteins combine incoming signals that are both positive and negative, and in response, they activate additional signaling regulators that are implicated in cancer, inflammatory pathways, cellular senescence, and stemness (101).

NLRs are PRRs that particularly recognize PAMPs, which were first studied as important regulators of immune response (102). Several gene mutations have been identified in NLRs, which render these proteins incapable of detecting PAMPs or self-assembly (102). By interfering with the NF-κB, MAPK, and/or caspase-1 signaling pathways, NLR variants with gain-of-function or loss-of-function mutations may contribute to the development of inflammatory disorders. The idea that innate immune signaling significantly contributes to the pathogenesis of endometriosis has also lately gained support due to accumulating evidence (103). Inflammatory signaling pathways may be taken over by endometriosis, leading to the proliferation, migration, and invasion of endometrial cells. However, the precise biochemical link between inflammation and endometriosis is still unknown. Since NLRs serve as signaling nodes in innate immunity, they are speculated to be potential therapeutic targets for treating endometriosis that is accompanied by inflammation.

### NLRC5 in endometriosis

**Figure 3:**
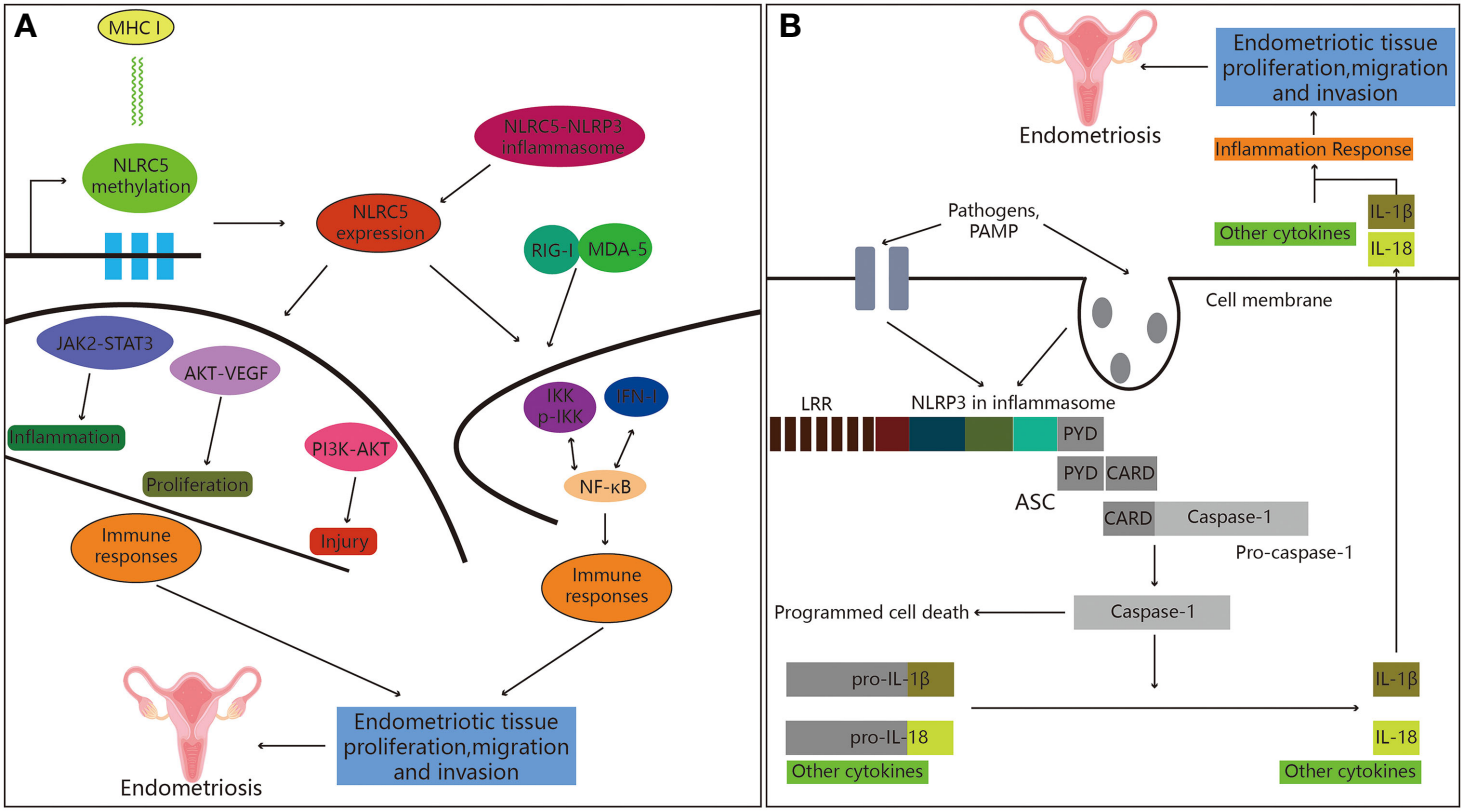
Role of NOD-like receptors in endometriosis. **(A)** NLRC5 and endometriosis. Firstly, the expression of the MHC class I gene is inversely associated with NLRC5 methylation. Secondly, inflammatory bodies can be created when NLRC5 and NLRP3 interact in the presence of pathogens. Finally, NLRC5 regulates immune response through a variety of signaling pathways that have been shown to play significant roles in the emergence of immune disorders, hence promoting the development of endometriosis. **(B)** Active NLRP3 inflammasome recruits ASC, followed by caspase-1 activation, IL-1β secretion and programmed cell death, leading to the development of endometriosis.

The innate immune molecule NLR family CARD domain containing 5 (NLRC5) is one of the highly conserved members of the newly discovered NLRs-like receptor family (104). Under the action of IFN-γ, two kinds of signaling transmitters and a transcriptional activator binding site on the promoter of NLRC5 bind to the phosphorylated STAT1 dimer, and the activated NLRC5 translocates to the nucleus (105) (**Figure 3A**). The translocation of NLRC5 from the cytoplasm to the nucleus also increases its regulatory function and scope (106, 107). Studies have reported that NLRC5 is widely involved in a variety of cellular processes, including immune, inflammatory and cell fate. In the immune response, NLRC5, as a major histocompatibility complex class I (MHC I) gene transactivation factor, activates the transcription of MHC I gene and the subsequent antigen presentation process (108, 109). Relevant studies have shown that NLRC5 is involved in mediating the immune escape of tumor cells, and activation of NLRC5 is known to inhibit tumor progression by promoting anti-tumor immune response (110, 111). During inflammatory response, NLRC5 may be involved as a negative regulator, suppressing inflammatory response by inhibiting the NF-κB inflammatory signaling pathway and the secretion of inflammatory factors (112). Notably, NLRC5 likely acts as a negative regulator in the development of endometriosis by inhibiting inflammation. By collecting clinical specimens of eutopic and ectopic endometrium from endometriosis patients, Zhan and co-workers found that the expression of NLRC5 in the above tissues from endometriosis patients was significantly higher than that in the normal endometrium (113). Mechanistically, NLRC5 inhibited inflammation by promoting autophagy through the extraction of secretory ectopic endometrial stromal cells (114). Therefore, these studies indicated that inflammatory conditions in endometriosis patients contributed to the elevated expression of NLRC5, where elevated levels of NLRC5 could suppress endometriosis by inhibiting inflammatory response.

### NLRP3 in endometriosis

NLR family pyrin domain containing 3 (NLRP3), a representative of the NLR family, is a type of extracellular receptor that detects exogenous and intrinsic danger signals (115). Previous studies have suggested that the development of endometriosis may be influenced by the NLRP3 inflammasome (57, 116). NLRP3, the adaptive protein apoptosis-related spotted protein has a caspase activation and recruitment domain (ASC), and along with caspase-1 (117), they make up the NLRP3 inflammasome (**Figure 3B**). The proteolytic enzyme, caspase-1, is involved in the process of pyroptosis (118). Gasdermin D is an executor of pyroptosis and required for IL-1β secretion (119). Pyroptotic cell death defends against intracellular pathogens in addition to its role in IL-1β release (120). NLRP3 inflammasome-mediated pyroptosis has been reported to play a significant role in the development of inflammatory diseases (121). Moreover, in a mouse model of endometriosis, they showed that the loss of NLRP3 reduced the bulk of endometrial pathology (122), and suppression of endometrial infiltration through the repression of NLRP3 activation and IL-1β generation in the mouse endometrial tissues, providing evidence for the involvement of the NLRP3 inflammasome in the pathogenesis of endometriosis (123). It was reported that estrogen promoted IL-1β production through estrogen receptors β mediated activation of the NLRP3 inflammasome in a murine model, which exacerbated the progression of inflammation and endometriosis, providing additional evidence for the role of NLRP3 inflammasome in endometriosis. Tripartite motif 24 ubiquitinated NLRP3, and its absence increased the activity of the NLRP3/Caspase-1/IL-1β-mediated pyroptosis signaling, which was suggested as the mechanism by which human endometrial stromal cells (hESCs) migrated and released the pro-inflammatory cytokines IL-1β and IL-18, promoting endometriosis (124). Targeted suppression of NLRP3 dramatically slowed the progression of endometriosis lesions and fibrogenesis in a mouse model of endometriosis (125). According to their research, mast cells were involved in the development of endometriosis through the activation of the NLRP3 inflammasome, which is known to be a nuclear-initiated estrogen signaling pathway. Progesterone suppresses the uptake of estrogen-induced NLRP3 inflammasome and IL-1β production through autophagy inducible inhibition. This study confirmed the anti-inflammatory function of autophagy in endometrial stromal cells (126). They also identified that the pathogenesis of endometriosis might be significantly supported by the imbalance in autophagy dependent NLRP3 inflammasome activation. NLRP3/IL-1β contributed to the etiology of endometriosis, and NLRP3 suppressors (MCC950) possibly helped to reduce ovarian endometriosis as well as enhanced the functionality of ovaries affected by endometriosis (127). These results indicated that NLRP3 suppressors may serve as a potential therapeutic target for the treatment of endometriosis.

## RLRs and endometriosis

### RLRs

Cytoplasmic nucleic acid receptors known as RLRs bind to RNA viruses, intermediary molecules in RNA replication, and transcription products (128). Retinoic acid-inducible gene I, melanoma differentiation factor 5 (MDA5) and laboratory of genetics and physiology 2 (LGP2) are the 3 components that constitute the RLRs family of receptors (129).

### Potential association between endometriosis and retinoic RLRs

**Figure 4:**
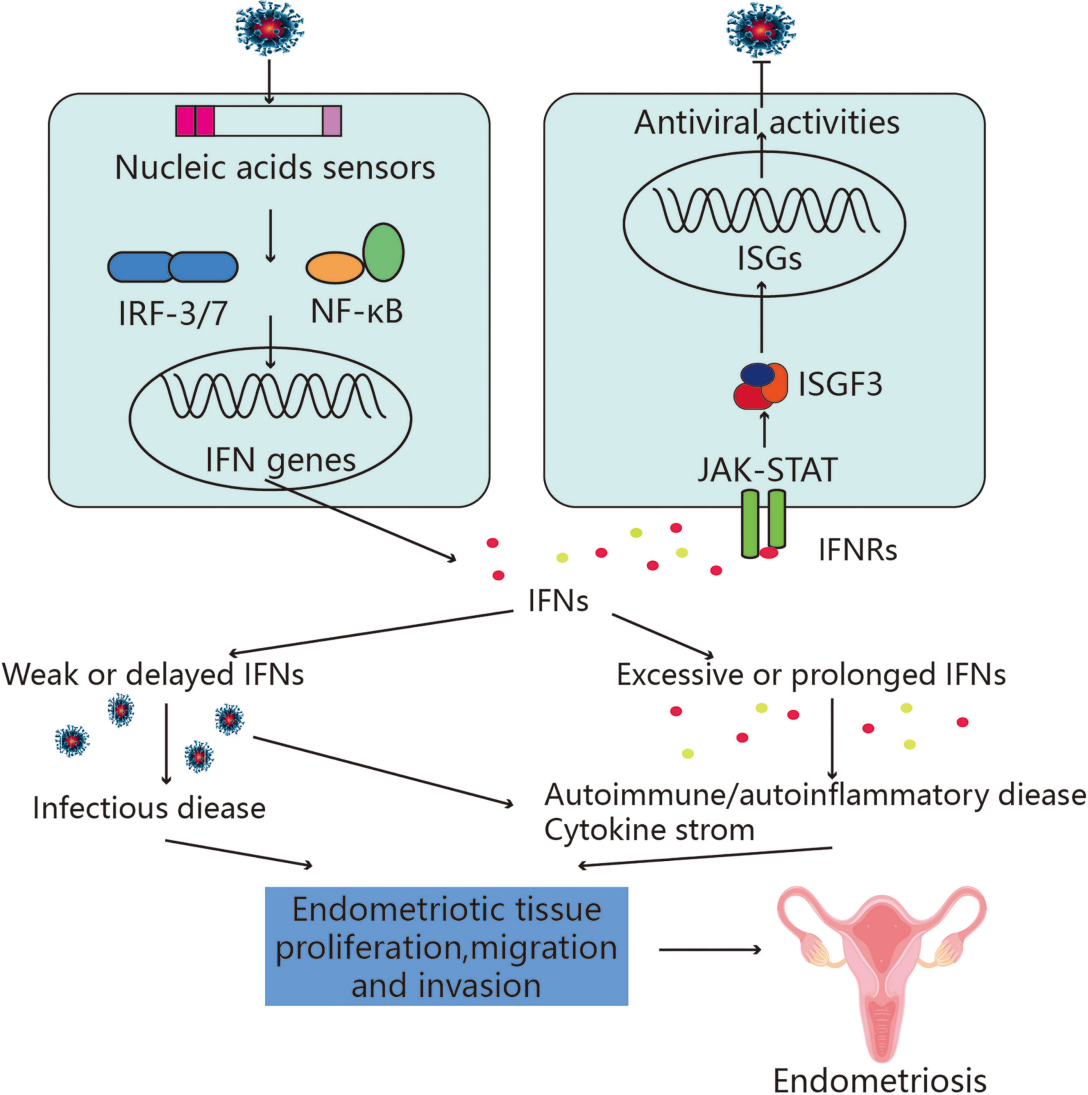
Potential association between RLRs and endometriosis. Nuclear translocation of the transcription activators IFN regulatory factor (IRF)3/7 and NF-κB and the detection of viral nucleic acids both intracellularly and extracellularly by nucleic acid sensors activate intracellular signaling pathways, which in turn activate the type I and type III interferon (IFN) genes. When type I and type III IFNs are secreted, they bind to their corresponding IFN receptor (IFNR) complexes in an autocrine and paracrine manner, activating a secondary signaling pathway. Strong antiviral responses are produced as a result of the downstream JAK-STAT pathway activating the transcription complex IFN-stimulated gene factor 3 (ISGF3), which in turn causes the production of hundreds of IFN-stimulated genes (ISGs). Since IFN-induced antiviral activity is essential for eradicating infectious viruses, a delayed or inadequate response causes the virus to spread unchecked. Interferon, on the other hand, is essential for controlling adaptive immunity. The development of autoimmune or autoimmune inflammatory illnesses, cytokine storms, and inflammation can all result from the excessive or chronic activation of interferon signaling, which may also be linked to the development and progression of endometriosis.

Mitochondrial antiviral-signaling (MAVS) protein is a key factor in the signal transduction pathway through RLRs receptors (130). During persistent viral infections and chronic immune activation, an elevated interferon signature and lymphoid tissue destruction correlate with disease progression. For example, in the context of a more physiologic HBV infection with a recombinant virus, HBV induced only a transient and modest increase in the expression of IFN and pro-inflammatory genes, which was associated with a persistent infection (131). When MAVS (**Figure 4**) is triggered, a giant signaling complex composed of TRAF protein and TBK1 (or IKKE (IκB kinase-E)) protein starts forming a prion-like filamentous structure. The ubiquitin chains attached to the TRAFs activate the IKK-a-IKKB-IKK-y triple complex. IRF3, IRF7, and/or NF-κB are subsequently activated as a result of this. IRF3, IRF7, and NF-κB boost the transcriptional activity of the genes such as the IFNs as well as other cytokines like TNF, IL-6, and IL-8 (108), with the help of AP-1. These inflammatory factors may be related to the development of endometriosis (132).

## ALRs and endometriosis

### ALRs

An extensive network of structurally related proteins known as ALRs are thought to function as intracellular DNA sensors, notifying the innate immune system when DNA is present on the cell membrane of infected or stressed cells (133, 134). Humans have 4 members in the ALR family (AIM2, interferon gamma-inducible 16 (IFI16), IFIX (PYHIN1), and MNDA), but mice have 13 members (including AIM2, p202, p203, p204, p205, p207, pyhin1) (135, 136). The inflammasome is also activated by proteins such as pyrin, AIM2, and IFI16. PRRs have received significant attention in recent years due to the variety of infectious and sterile stimuli that they can respond to, implicating them in a variety of disorders (137, 138). Pyroptosis, an inflammatory version of cell death, always occurs when the inflammasome is activated (139). Due to the nature of this type of cell death, pyrogenesis in the affected cells leads to the eradication of the etiological sites but is associated with tissue damage (140). We speculate that ALRs may trigger the inflammasome to promote endometriosis development.

### Potential association between endometriosis and ALRs

**Figure 5:**
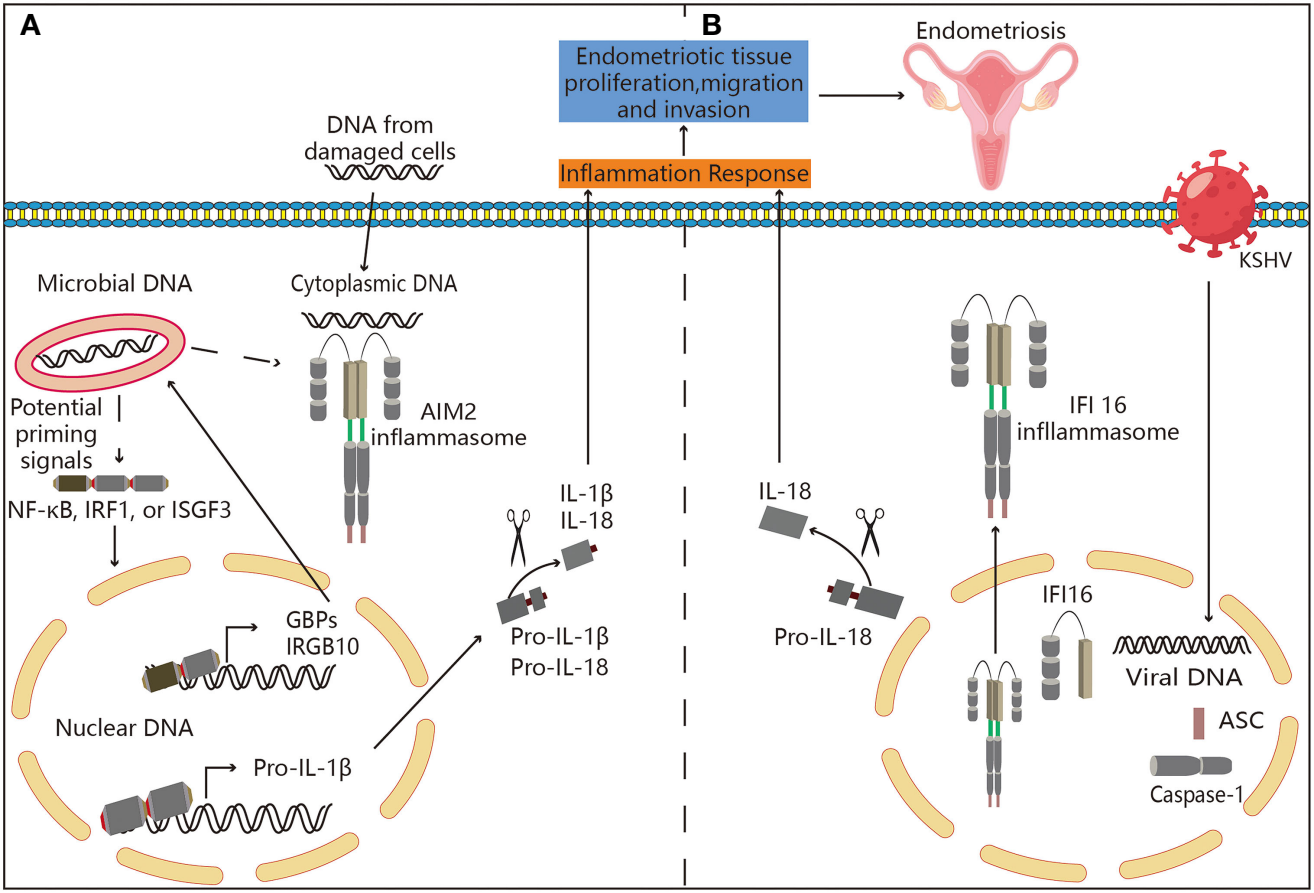
Potential association between ALRs and endometriosis. **(A)** Mechanisms associated with AIM2 inflammasome activation. AIM2 is present in an inactive state in the cell’s cytoplasm. AIM2 acquires an open, active conformation after binding to cytoplasmic DNA in a sequence independent manner. This conformation enables attachment to the adaptor protein ASC, which binds to caspase-1. This entire inflammasome enables caspase-1 activation, proteolytic processing, and cytokine IL-1 and IL-18 activation. Some diseases require the release of their DNA into the cytoplasm from inside the pathogen itself. The type-I interferon response induces guanylate-binding proteins (GBPs) and IRGB10. In order to enable the release of pathogenic DNA into the cytoplasm, where it can activate AIM2, GBPs and IRGB10 bind to pathogens and break down their membranes. Thus, AIM2 may be involved in the pathogenesis of endometriosis. **(B)** Inflammasome is assembled in the nucleus by IFI16. IFI16’s recognition of KSHV genomes results in the assembly of nuclear IFI16 inflammasomes. The formation and development of endometriosis may be linked to the inflammasome’s translocation to the nucleus, and then it cleaves pro-IL-18 into its physiologically active version.

ALRs are present in the cytoplasm in its inactive conformation (141). AIM2 (**Figure 5A**) acquires an open, active conformation upon association with cytoplasmic DNA in a sequence-independent manner. This conformation allows it to attach with the adaptor protein ASC, which binds to caspase-1 (142). As a result, there is a whole inflammasome that enables the function of caspase-1 and proteolytic processing as well as activates the cytokines IL-1 and IL-18 (143). Some diseases are characterized by the release of DNA from the pathogen into the cytoplasm. The type-I interferon response induces the expression of guanylate-binding proteins (GBPs) and IRGB10. In order to enable the pathogenic DNA to be released into the cytoplasm, where AIM2 can be triggered, GBPs and IRGB10 combine with the pathogens and break their membranes (144). Overall, activation of the AIM2 inflammasome appears to be critical for host protection as well as clearance of intracellular pathogens such as bacteria, viruses, fungi and parasites (145). AIM2 activation or some pathogen and host encoded modulators may also regulate pathogen proliferation, inflammation, and tissue damage (146). Therefore, further research on the implications of AIM2 inflammasome activation are necessary. The IFN-inducible p200 (also known as PYHIN) family, of which IFI16 is a member, is primarily composed of nuclear proteins (147). Assembly of the nuclear IFI16 (**Figure 5B**) inflammasome is triggered by IFI16’s recognition of the KSHV genomes (148). Upon sensing DNA, cytosolic and nuclear (IFI16) PYHIN proteins recruit an adaptor protein (ASC) and pro-caspase-1 to form an inflammasome, which activates caspase-1. Activated caspase-1 then cleaves pro-IL-1β and pro-IL-18 to generate their active forms (149). In earlier studies (150, 151), IFI16 has been implicated in the regulation of the cell cycle, differentiation and apoptosis, which are explained in greater detail elsewhere. Inflammasome-mediated pyroptosis has been reported to play an important role in the development of inflammatory diseases. We hypothesiz that loss of ALRs reduces the volume of endometrial pathology and inhibits endometrial infiltration by inhibiting the inactivation of ALRs and IL-1β production in endometrial tissues, which is a possible mechanism for the involvement of ALRs inflammasome in the pathogenesis of endometriosis. However, animal studies or clinical evidence showing a specific link between ALR inflammasome and endometriosis is lacking.

## Potential clinical applications of PRRs for treating endometriosis

Several modern treatment options are currently available for the management of endometriosis symptoms (152). Several arguments can be made for the use of medical treatment in endometriosis for lifelong management. For example, both hormonal and Non-steroidal anti-inflammatory medications (NSAID) treatments decrease inflammation, which is a key aspect of the pathogenesis of endometriosis. However, all these therapies serve as suppressive measures rather than therapeutic ones. Endometriomas, deeply infiltrating diseases, and increased fecundity cannot be treated with the currently available medical interventions (153). NSAIDs and hormone therapy have served as the cornerstones of conventional endometriosis treatment. The most widely used hormonal medications include a combined oral contraceptives (COCs), progestogens, gonadotropin-releasing hormone (GnRH) agonists, androgens, and anti-progestogens (154, 155). All of them are thought to have comparable effectiveness but differing, sometimes unfavorable, tolerability profiles. Because pain has the greatest influence on the quality of life of endometriosis patients and is the condition for which novel medical treatments are most urgently needed, we focused on medications based on their pain reduction ratings as the key endpoint (156). Currently, there are a few medical treatments available for patients seeking relief from discomfort symptoms, especially those who are trying to get pregnant (157). We are aware of this unmet need and have reassessed our approach for the development of novel therapeutics by focusing on processes like inflammation and pertinent receptors or signaling pathways implicated in the production of pain symptoms. This was achieved by taking advantage of new and emerging information on the pathophysiology of the disorder. Several registered clinical studies exploring the efficacy of new pharmacological therapies have considered endometriosis-related pelvic discomfort as one of the major inclusion criteria for the patients (**Table 3**). We identified that medications which were frequently prescribed for endometriosis in medical settings could also affect other illnesses by acting on PRRs. The discovery that aspirin could influence other disease processes through PRRs caught us by surprise. Aspirin, for instance, protects against acute kidney injury caused by lipopolysaccharide through the TLR4/MyD88/NF-κB pathway (158). At the same time, it was shown that aspirin-triggered lipoxin reduced cerebral infarction through the regulation of TLR4/NF-κB-mediated endoplasmic reticulum stress in a mouse model (159). Indobufen, aspirin, and their combinations with clopidogrel or ticagrelor reduced the symptoms of inflammasome-mediated pyroptosis in ischemic stroke by blocking the NF-κB/NLRP3 signaling pathway (160). Progesterone induced blocking factor (PIBF), which was identified as a possible therapeutic target, has been reported to be involved in some leukemia patients to evade immune monitoring. Progesterone, through its effects on the LPS receptor, TLR signaling, and antimicrobial peptides, may affect infection and autoimmune disease progression (168). Progesterone inhibits interferon signaling by suppressing the expression of TLR7 and the myxovirus resistance protein A in the peripheral blood mononuclear cells of hepatitis C virus-infected patients (161). Progesterone plus vitamin D treatment reduced inflammation after traumatic brain damage and did so by modulating the TLR4/NF-κB signaling pathway (162). Progesterone also protected against Aβ-induced NLRP3-Caspase-1 inflammasome activation by increasing autophagy in astrocytes (163). Progesterone was shown to reduce stress-induced NLRP3 inflammasome activation and increase autophagy after ischemic brain injury (164). In monocytes from preeclampsia-affected pregnant women, progesterone and vitamin D inhibited the activation of the TLR4-MyD88-NF-κB pathway and the NLRP1/NLRP3 inflammasomes (165). In endometrial epithelial cells, LPS and HMGB-1 stimulated TLR4 expression, which was inhibited by dienogest (169). Blocking corticosterone activity with the glucocorticoid receptor antagonist mifepristone, suppressed the elevation of NLRP3 and HMGB1 in unchallenged rats, regulated the proinflammatory response to LPS, and prevented memory impairment (166). Mifepristone exerted protective effects against NLRP1 inflammasome activation and prolonged dexamethasone-induced damage to hippocampus neurons (167). Therefore, strategies to block the NLRP1 inflammasome axis may serve as potential therapeutic options for the treatment of endometriosis.

**Table 3:** Potential clinical applications of pattern recognition receptors in treating endometriosis.

Durg	Study population	Primary outcome	Route	Trial number	Status	PRRs
Aspirin	endometriosis-associated pelvic pain	pain score (overall pain)	oral	NCT05156879	phase IV-recruiting	Aspirin protects against acute kidney injury caused by lipopolysaccharide through the TLR4/MyD88/NF-κB pathway (158). Aspirin-triggered lipoxin reduced cerebral infarction through the regulation of TLR4/NF-κB-mediated endoplasmic reticulum stress in a mouse model (159). Indobufen, aspirin, and their combinations with clopidogrel or ticagrelor reduced the symptoms of inflammasome-mediated pyroptosis in ischemic stroke by blocking the NF-κB/NLRP3 signaling pathway (160).
Contraceptive	endometriosis-associated pain	patients' satisfaction (pain reduction and adverse side effects)	intramuscular(oral)	NCT01056042	phase IV-completed	
Contraceptive (versus leuprolide/norethindrone)	endometriosis-associated pelvic pain	pain and quality of life	oral	NCT00229996	phase III-completed	
Medroxyprogesterone(versus levonorgestrel-intrauterine system)	endometriosis-associated pelvic pain	pain score (severity of pelvic pain)	oral (intrauterine system)	NCT02534688	phase IV-completed	Progesterone inhibits interferon signaling by suppressing the expression of TLR7 and the myxovirus resistance protein A in the peripheral blood mononuclear cells of hepatitis C virus-infected patients (161). Progesterone plus vitamin D treatment reduced inflammation after traumatic brain damage and does so by modulating the TLR4/NF-κB signaling pathway (162). Progesterone also protected against Aβ-induced NLRP3-Caspase-1 inflammasome activation by increasing autophagy in astrocytes (163). Progesterone reduce stress-induced NLRP3 inflammasome activation and increase autophagy after ischemic brain injury (164). In monocytes from preeclampsia-affected pregnant women, progesterone and vitamin D inhibited the activation of the TLR4-MyD88-NF-κB pathway and the NLRP1/NLRP3 inflammasomes (165).
Dienogest	confirmed of endometriosis	pain score (severity of pelvic pain)	oral	NCT01822080	phase III-completed	In endometrial epithelial cells, LPS and HMGB-1 stimulated TLR4 expression, which was inhibited by dienogest
Dienogest (versus oral contraceptive pills)	confirmed of endometriosis; painful symptoms	pain score (severity of pelvic pain)	oral	NCT04256200	phase II-recruiting; phase III-recruiting	
Dienogest (Visanne, BAY86-5258)	confirmed of endometriosis	pain-related quality of Life	oral	NCT02425462	prospective study-completed	
Mifepristone	confirmed of endometriosis; painful symptoms	dysmenorrhea (changes and intensity)	oral	NCT02271958	phase II-completed; phase III-completed	Blocking corticosterone activity with the glucocorticoid receptor antagonist mifepristone, suppressed the elevation of NLRP3 and HMGB1 in unchallenged rats, regulated the proinflammatory response to LPS, and prevented memory impairment (166). Mifepristone exerted protective effects against NLRP1 inflammasome activation and prolonged dexamethasone-induced damage to hippocampus neurons (167).
Danazol	confirmed of endometriosis; painful symptoms	pain associated with endometriosis	oral	NCT00758953	phase II-completed	
Leuprolide (plus anastrazole)	endometriosis recurrence treatment	Disease free time (time of pain disappearance and reduction of endometriosis lesion)	oral	NCT01769781	phase IV-completed	
Elagolix	moderate to severe endometriosis-associated pain in adult premenopausal female	pain score (overall pain)	oral	NCT01620528 NCT01760954 NCT01931670 NCT02143713	phase IV-completed	

As mentioned above, medications frequently used in endometriosis treatment may also be effective against other diseases due to their action on PRRs. Meanwhile, given the significant roles that PRRs play in the pathophysiology and progression of many diseases, they may be useful therapeutic targets (43). However, there may be a safety concern because many of these receptors regulate NF-κB and IRF activation, similar to other PRRs, and their inhibition may interfere with host immune response to infection (170). It makes sense to look at potential targets upstream of this event that may decrease the activity of some PRRs while preserving the ability of other PRRs that respond to infection. Additionally, inflammation and neurological pathways also have a role in the development of endometriosis-related pelvic pain (171). Closely associated with pain induced by neuronal pathways are PRRs, particularly TLR receptors (172–174). Lidocaine affects nerve terminals and intraperitoneal macrophages in addition to having anti-inflammatory characteristics (175, 176). A double-blind, randomized, phase-II clinical trial was conducted to assess the effect of lidocaine on endometriosis patients with severe dysmenorrhea. Based on this randomized controlled trial, patients with endometriosis and dysmenorrhea may benefit from treatment with lidocaine as a non-hormonal therapy (177). In LPS-stimulated murine macrophages, lidocaine strongly suppressed the activation of TLR4, NF-κB, and MAPKs (178). In a mouse model of allergic airway inflammation, lidocaine reduced allergic airway inflammation through TLR2, indicating that the TLR2/NF-κB/NLRP3 pathway may provide a promising therapeutic approach for treating allergic airway inflammation (179). In summary, we speculate that indobufen, aspirin, and their combinations with clopidogrel or ticagrelor may find several potential applications for targeting PRRs in the treatment of endometriosis.

## Discussion

The functions of PRRs in endometriosis have been outlined in this review. Suppression of hyperinflammatory response and controlling regular infectious agents could be used as a potential therapeutic target for the treatment of endometriosis. Although experience suggests that the PRRs-mediated immune and inflammatory responses are involved in endometriosis, additional research is needed for clarifying the following issues:

(1) The role of RLRs and ALRs in endometriosis: There has been a huge progress in our understanding of the role of TLRs, NLRs, and CLRs in endometriosis. Other PRRs, such as RLRs and ALRs are also important immune response initiators, similar to TLRs, NLRs, and CLRs. However, their functions in endometriosis are still unknown. Better understanding of the function of the above receptors in endometriosis would enable the discovery of novel therapeutic targets in endometriosis and help improve the clinical symptoms of endometriosis patients.

(2) PRRs produced from non-immune cells and their functions in endometriosis: A great deal of research has been done to identify the function of immune cell-derived PRRs in endometriosis. However, there is accumulating evidence that the immune response to endometriosis is also heavily dependent on the expression of PRRs that are expressed in non-immune cells, such as endothelial and epithelial cells. Nevertheless, additional studies are necessary in order to fully understand their function in endometriosis.

(3) Creation of brand-new PRRs antagonists and inhibitors: Endometriosis treatment strategies may change if PRRs and the signaling proteins they interact with are targeted. Sadly, there have been incredibly few clinical trials on the use of PRRs inhibitors and antagonists for the management of endometriosis symptoms. The creation of novel PRRs inhibitors and antagonists, as well as the confirmation of their therapeutic functions in endometriosis, may represent a viable approach for the development of new therapeutic strategies for treating endometriosis.

## Author contributions

BG and LZ designed the study and edited the final text. BG, JC, JHZ and YF collected the data from publications, developed the database and wrote the manuscript. BG prepared the figures. BG, XL and JZ prepared the tables. BG, JC, JHZ, YF XL, JZ, HZ and LZ contributed to the manuscript revision and critical discussion. All authors contributed to the article and approved the submitted version.

## References

[1] Zondervan KT Becker CM Missmer SA. Endometriosis. N Engl J Med (2020) 382(13):1244–56. doi: 10.1056/NEJMra1810764 32212520

[2] Aghajanova L Tatsumi K Horcajadas JA Zamah AM Esteban FJ Herndon CN. Unique transcriptome, pathways, and networks in the human endometrial fibroblast response to progesterone in endometriosis. Biol Reprod (2011) 84(4):801–15. doi: 10.1095/biolreprod.110.086181 PMC3062042 20864642

[3] Cheng Y-H Imir A Fenkci V Yilmaz MB Bulun SE. Stromal cells of endometriosis fail to produce paracrine factors that induce epithelial 17 beta-hydroxysteroid dehydrogenase type 2 gene and its transcriptional regulator Sp1: A mechanism for defective estradiol metabolism. Am J Obstetrics Gynecology (2007) 196(4):391.e1–7–391.e7–8. doi: 10.1016/j.ajog.2006.12.014 17403431

[4] Cheng Y-H Yin P Xue Q Yilmaz B Dawson MI Bulun SE. Retinoic acid (Ra) regulates 17 beta-hydroxysteroid dehydrogenase type 2 expression in endometrium: Interaction of Ra receptors with specificity protein (Sp) 1/Sp3 for estradiol metabolism. J Clin Endocrinol Metab (2008) 93(5):1915–23. doi: 10.1210/jc.2007-1536 PMC2386682 18270252

[5] Bulun SE Yilmaz BD Sison C Miyazaki K Bernardi L Liu S. Endometriosis. Endocrine Rev (2019) 40(4):1048–79. doi: 10.1210/er.2018-00242 PMC6693056 30994890

[6] Golabek-Grenda A Olejnik A. *In vitro* modeling of endometriosis and endometriotic microenvironment - challenges and recent advances. Cell Signalling (2022) 97:110375. doi: 10.1016/j.cellsig.2022.110375 35690293

[7] Zhang XM Odom DT Koo SH Conkright MD Canettieri G Best J. Genome-wide analysis of camp-response element binding protein occupancy, phosphorylation, and target gene activation in human tissues. Proc Natl Acad Sci United States America (2005) 102(12):4459–64. doi: 10.1073/pnas.0501076102 PMC555478 15753290

[8] Kvaskoff M Mahamat-Saleh Y Farland LV Shigesi N Terry KL Harris HR. Endometriosis and cancer: A systematic review and meta-analysis. Hum Reprod Update (2021) 27(2):393–420. doi: 10.1093/humupd/dmaa045 33202017

[9] Della Corte L Di Filippo C Gabrielli O Reppuccia S La Rosa VL Ragusa R. The burden of endometriosis on women's lifespan: A narrative overview on quality of life and psychosocial wellbeing. . Int J Environ Res Public Health (2020) 17(13):4683. doi: 10.3390/ijerph17134683 32610665 PMC7370081

[10] Asghari S Valizadeh A Aghebati-Maleki L Nouri M Yousefi M. Endometriosis: Perspective, lights, and shadows of etiology. BioMed Pharmacother (2018) 106:163–74. doi: 10.1016/j.biopha.2018.06.109 29958140

[11] Zakhari A Delpero E McKeown S Tomlinson G Bougie O Murji A. Endometriosis recurrence following post-operative hormonal suppression: A systematic review and meta-analysis. Hum Reprod Update (2021) 27(1):96–107. doi: 10.1093/humupd/dmaa033 33020832 PMC7781224

[12] Taylor HS Kotlyar AM Flores VA. Endometriosis is a chronic systemic disease: Clinical challenges and novel innovations. Lancet (2021) 397(10276):839–52. doi: 10.1016/s0140-6736(21)00389-5 33640070

[13] Lee Y Lee Y Lee S Jung S Chon S. Correlation of preoperative biomarkers with severity of adhesion in endometriosis. J Gynecology Obstetrics Hum Reprod (2020) 49(1):101637. doi: 10.1016/j.jogoh.2019.101637 31520750

[14] Laschke MW Menger MD. Basic mechanisms of vascularization in endometriosis and their clinical implications. Hum Reprod Update (2018) 24(2):207–24. doi: 10.1093/humupd/dmy001 29377994

[15] Sampson JA. Metastatic or embolic endometriosis, due to the menstrual dissemination of endometrial tissue into the venous circulation. Am J Pathol (1927) 3(2):93–110. doi: 10.1007/BF01995231 19969738 PMC1931779

[16] Yovich JL Rowlands PK Lingham S Sillender M Srinivasan S. Pathogenesis of endometriosis: Look no further than John Sampson. Reprod BioMed Online (2020) 40(1):7–11. doi: 10.1016/j.rbmo.2019.10.007 31836436

[17] Symons LK Miller JE Kay VR Marks RM Liblik K Koti M. The immunopathophysiology of endometriosis. Trends Mol Med (2018) 24(9):748–62. doi: 10.1016/j.molmed.2018.07.004 30054239

[18] Izumi G Koga K Takamura M Makabe T Satake E Takeuchi A. Involvement of immune cells in the pathogenesis of endometriosis. J Obstet Gynaecol Res (2018) 44(2):191–8. doi: 10.1111/jog.13559 29316073

[19] Machairiotis N Vasilakaki S Thomakos N. Inflammatory mediators and pain in endometriosis: A systematic review. Biomedicines (2021) 9(1):54. doi: 10.3390/biomedicines9010054 33435569 PMC7826862

[20] Moghaddam MZ Ansariniya H Seifati SM Zare F Fesahat F. Immunopathogenesis of endometriosis: An overview of the role of innate and adaptive immune cells and their mediators. Am J Reprod Immunol (2022) 87(5):e13537. doi: 10.1111/aji.13537 35263479

[21] Sanchez AM Vanni VS Bartiromo L Papaleo E Zilberberg E Candiani M. Is the oocyte quality affected by endometriosis? A review of the literature. J Ovarian Res (2017) 10(1):43. doi: 10.1186/s13048-017-0341-4 28701212 PMC5508680

[22] Hey-Cunningham AJ Wong C Hsu J Fromm PD Clark GJ Kupresanin F. Comprehensive analysis utilizing flow cytometry and immunohistochemistry reveals inflammatory changes in local endometrial and systemic dendritic cell populations in endometriosis. Hum Reprod (2021) 36(2):415–28. doi: 10.1093/humrep/deaa318 33313846

[23] Maddern J Grundy L Castro J Brierley SM. Pain in endometriosis. Front Cell Neurosci (2020) 14:590823. doi: 10.3389/fncel.2020.590823 33132854 PMC7573391

[24] Patel BG Lenk EE Lebovic DI Shu Y Yu J Taylor RN. Pathogenesis of endometriosis: Interaction between endocrine and inflammatory pathways. Best Pract Res Clin Obstet Gynaecol (2018) 50:50–60. doi: 10.1016/j.bpobgyn.2018.01.006 29576469

[25] Kolanska K Alijotas-Reig J Cohen J Cheloufi M Selleret L d'Argent E. Endometriosis with infertility: A comprehensive review on the role of immune deregulation and immunomodulation therapy. Am J Reprod Immunol (2021) 85(3):e13384. doi: 10.1111/aji.13384 33278837

[26] Crispim PCA Jammal MP Murta EFC Nomelini RS. Endometriosis: What is the influence of immune cells? Immunol Invest (2021) 50(4):372–88. doi: 10.1080/08820139.2020.1764577 32408782

[27] Chen S Chai X Wu X. Bioinformatical analysis of the key differentially expressed genes and associations with immune cell infiltration in development of endometriosis. BMC Genom Data (2022) 23(1):20. doi: 10.1186/s12863-022-01036-y 35303800 PMC8932180

[28] Giacomini E Minetto S Li Piani L Pagliardini L Somigliana E Vigano P. Genetics and inflammation in endometriosis: Improving knowledge for development of new pharmacological strategies. Int J Mol Sci (2021) 22(16):9033. doi: 10.3390/ijms22169033 34445738 PMC8396487

[29] Kumar H Kawai T Akira S. Pathogen recognition by the innate immune system. Int Rev Immunol (2011) 30(1):16–34. doi: 10.3109/08830185.2010.529976 21235323

[30] Chen G Shaw MH Kim YG Nunez G. Nod-like receptors: Role in innate immunity and inflammatory disease. Annu Rev Pathol (2009) 4:365–98. doi: 10.1146/annurev.pathol.4.110807.092239 18928408

[31] Yeo SG Won YS Kim SH Park DC. Differences in c-type lectin receptors and their adaptor molecules in the peritoneal fluid of patients with endometriosis and gynecologic cancers. Int J Med Sci (2018) 15(4):411–6. doi: 10.7150/ijms.23360 PMC5835712 29511377

[32] Wu X-G Chen J-J Zhou H-L Wu Y Lin F Shi J. Identification and validation of the signatures of infiltrating immune cells in the eutopic endometrium endometria of women with endometriosis. Front Immunol (2021) 12:671201. doi: 10.3389/fimmu.2021.671201 34539624 PMC8446207

[33] Onomoto K Onoguchi K Yoneyama M. Regulation of rig-I-Like receptor-mediated signaling: Interaction between host and viral factors. Cell Mol Immunol (2021) 18(3):539–55. doi: 10.1038/s41423-020-00602-7 PMC7812568 33462384

[34] Lupfer CR Rippee-Brooks MD Anand PK. Common differences: The ability of inflammasomes to distinguish between self and pathogen nucleic acids during infection. Int Rev Cell Mol Biol (2019) 344:139–72. doi: 10.1016/bs.ircmb.2018.10.001 30798987

[35] Brubaker SW Bonham KS Zanoni I Kagan JC. Innate immune pattern recognition: A cell biological perspective. Annu Rev Immunol (2015) 33:257–90. doi: 10.1146/annurev-immunol-032414-112240 PMC5146691 25581309

[36] Kawai T Akira S. Toll-like receptors and their crosstalk with other innate receptors in infection and immunity. Immunity (2011) 34(5):637–50. doi: 10.1016/j.immuni.2011.05.006 21616434

[37] Mnich ME van Dalen R van Sorge NM. C-type lectin receptors in host defense against bacterial pathogens. Front Cell Infection Microbiol (2020) 10:309. doi: 10.3389/fcimb.2020.00309 PMC7358460 32733813

[38] Kim YK Shin J-S Nahm MH. Nod-like receptors in infection, immunity, and diseases. Yonsei Med J (2016) 57(1):5–14. doi: 10.3349/ymj.2016.57.1.5 26632377 PMC4696971

[39] Iurescia S Fioretti D Rinaldi M. Targeting cytosolic nucleic acid-sensing pathways for cancer immunotherapies. Front Immunol (2018) 9:711. doi: 10.3389/fimmu.2018.00711 29686682 PMC5900005

[40] Caneparo V Landolfo S Gariglio M De Andrea M. The absent in melanoma 2-like receptor ifn-inducible protein 16 as an inflammasome regulator in systemic lupus erythematosus: The dark side of sensing microbes. Front Immunol (2018) 9:1180. doi: 10.3389/fimmu.2018.01180 29892303 PMC5985366

[41] Zindel J Kubes P. Damps, pamps, and lamps in immunity and sterile inflammation. Annu Rev Pathology: Mech Dis (2020) 15:493–518. doi: 10.1146/annurev-pathmechdis-012419-032847 31675482

[42] Saijo Y EPi L Yasuda S. Pattern recognition receptors and signaling in plant–microbe interactions. Plant J (2018) 93(4):592–613. doi: 10.1111/tpj.13808 29266555

[43] Gong T Liu L Jiang W Zhou R. Damp-sensing receptors in sterile inflammation and inflammatory diseases. Nat Rev Immunol (2020) 20(2):95–112. doi: 10.1038/s41577-019-0215-7 31558839

[44] He S He J Chen J Chen F Ou S. Research advancement of innate immunity and pattern recognition receptors. Chin J Anim Nutr (2017) 29(11):3844–51.

[45] Mortaz E Adcock IM Tabarsi P Darazam IA Movassaghi M Garssen J. Pattern recognitions receptors in immunodeficiency disorders. Eur J Pharmacol (2017) 808:49–56. doi: 10.1016/j.ejphar.2017.01.014 28095323

[46] Li D Wu M. Pattern recognition receptors in health and diseases. Signal Transduct Target Ther (2021) 6(1):291. doi: 10.1038/s41392-021-00687-0 34344870 PMC8333067

[47] Tominari T Akita M Matsumoto C Hirata M Yoshinouchi S Tanaka Y. Endosomal Tlr3 signaling in stromal osteoblasts induces prostaglandin e-2-Mediated inflammatory periodontal bone resorption. J Biol Chem (2022) 298(3):101603. doi: 10.1016/j.jbc.2022.101603 35101442 PMC8892075

[48] Deswaerte V Ruwanpura SM Jenkins BJ. Transcriptional regulation of inflammasome-associated pattern recognition receptors, and the relevance to disease pathogenesis. Mol Immunol (2017) 86:3–9. doi: 10.1016/j.molimm.2016.09.023 27697299

[49] Deretic V Saitoh T Akira S. Autophagy in infection, inflammation and immunity. Nat Rev Immunol (2013) 13(10):722–37. doi: 10.1038/nri3532 PMC5340150 24064518

[50] Tan X Sun L Chen J Chen ZJ. Detection of microbial infections through innate immune sensing of nucleic acids. Annu Rev Microbiol (2018) 72:447–78. doi: 10.1146/annurev-micro-102215-095605 30200854

[51] Palm NW Medzhitov R. Pattern recognition receptors and control of adaptive immunity. Immunol Rev (2009) 227:221–33. doi: 10.1111/j.1600-065X.2008.00731.x 19120487

[52] Kobayashi H Higashiura Y Shigetomi H Kajihara H. Pathogenesis of endometriosis: The role of initial infection and subsequent sterile inflammation (Review). Mol Med Rep (2014) 9(1):9–15. doi: 10.3892/mmr.2013.1755 24173432

[53] Kawai T Akira S. The roles of tlrs, rlrs and nlrs in pathogen recognition. Int Immunol (2009) 21(4):317–37. doi: 10.1093/intimm/dxp017 PMC2721684 19246554

[54] Akira S Uematsu S Takeuchi O. Pathogen recognition and innate immunity. Cell (2006) 124(4):783–801. doi: 10.1016/j.cell.2006.02.015 16497588

[55] Trinchieri G Sher A. Cooperation of toll-like receptor signals in innate immune defence. Nat Rev Immunol (2007) 7(3):179–90. doi: 10.1038/nri2038 17318230

[56] de Azevedo BC Mansur F Podgaec S. A systematic review of toll-like receptors in endometriosis. Arch Gynecology Obstetrics (2021) 304(2):309–16. doi: 10.1007/s00404-021-06075-x 33928453

[57] Bullon P Manuel Navarro J. Inflammasome as a key pathogenic mechanism in endometriosis. Curr Drug Targets (2017) 18(9):997–1002. doi: 10.2174/1389450117666160709013850 27397068

[58] Sikora J Mielczarek-Palacz A Kondera-Anasz Z. Imbalance in cytokines from interleukin-1 family - role in pathogenesis of endometriosis. Am J Reprod Immunol (2012) 68(2):138–45. doi: 10.1111/j.1600-0897.2012.01147.x 22537218

[59] Akoum A Al-Akoum M Lemay A Maheux R Leboeuf M. Imbalance in the peritoneal levels of interleukin 1 and its decoy inhibitory receptor type ii in endometriosis women with infertility and pelvic pain. Fertility Sterility (2008) 89(6):1618–24. doi: 10.1016/j.fertnstert.2007.06.019 17919610

[60] Zhang Y Liang X Bao X Xiao W Chen G. Toll-like receptor 4 (Tlr4) inhibitors: Current research and prospective. Eur J Med Chem (2022) 235:114291. doi: 10.1016/j.ejmech.2022.114291 35307617

[61] Hu J Xu J Feng X Li Y Hua F Xu G. Differential expression of the Tlr4 gene in pan-cancer and its related mechanism. Front Cell Dev Biol (2021) 9:700661. doi: 10.3389/fcell.2021.700661 34631699 PMC8495169

[62] Noreen M Arshad M. Association of Tlr1, Tlr2, Tlr4, Tlr6, and tirap polymorphisms with disease susceptibility. Immunol Res (2015) 62(2):234–52. doi: 10.1007/s12026-015-8640-6 25784622

[63] Ibanez F Montesinos J Urena-Peralta JR Guerri C Pascual M. Tlr4 participates in the transmission of ethanol-induced neuroinflammation *Via* astrocyte-derived extracellular vesicles. J Neuroinflamm (2019) 16(1):136. doi: 10.1186/s12974-019-1529-x PMC6610989 31272469

[64] Lupi LA Cucielo MS Silveira HS Gaiotte LB Cesario RC Seiva FRF. The role of toll-like receptor 4 signaling pathway in ovarian, cervical, and endometrial cancers. Life Sci (2020) 247:117435. doi: 10.1016/j.lfs.2020.117435 32081661

[65] Chen Y Zhao YF Yang J Jing HY Liang W Chen MY. Selenium alleviates lipopolysaccharide-induced endometritis *Via* regulating the recruitment of Tlr4 into lipid rafts in mice. Food Funct (2020) 11(1):200–10. doi: 10.1039/c9fo02415h 31845693

[66] Rashidi N Mirahmadian M Tehrani MJ Rezania S Ghasemi J Kazemnejad S. Lipopolysaccharide- and lipoteichoic acid-mediated pro-inflammatory cytokine production and modulation of Tlr2, Tlr4 and Myd88 expression in human endometrial cells. J Reprod Infertility (2015) 16(2):72–81. doi: 10.2307/3602384 PMC4386089 25927023

[67] Koval H Chopiak V. Mrna Tlr2 and Tlr4 expression in the endometrium tissue in women with endometriosis assosiated with infertility. Georgian Med News (2015) 244-245):7–11. 26177128

[68] Azuma Y Taniguchi F Nakamura K Nagira K Khine YM Kiyama T. Lipopolysaccharide promotes the development of murine endometriosis-like lesions *Via* the nuclear factor-kappa b pathway. Am J Reprod Immunol (2017) 77(4):e12631. doi: 10.1111/aji.12631 28138997

[69] Khan KN Kitajima M Hiraki K Yamaguchi N Katamine S Matsuyama T. Escherichia coli contamination of menstrual blood and effect of bacterial endotoxin on endometriosis. Fertility sterility (2010) 94(7):2860–3. e3. doi: 10.1016/j.fertnstert.2010.04.053 20627244

[70] Kajihara H Yamada Y Kanayama S Furukawa N Noguchi T Haruta S. New insights into the pathophysiology of endometriosis: From chronic inflammation to danger signal. Gynecol Endocrinol (2011) 27(2):73–9. doi: 10.3109/09513590.2010.507292 20712428

[71] Bora G Yaba A. The role of mitogen-activated protein kinase signaling pathway in endometriosis. J Obstetrics Gynaecology Res (2021) 47(5):1610–23. doi: 10.1111/jog.14710 33590617

[72] Kutikhin AG. Association of polymorphisms in tlr genes and in genes of the toll-like receptor signaling pathway with cancer risk. Hum Immunol (2011) 72(11):1095–116. doi: 10.1016/j.humimm.2011.07.307 21872627

[73] Jafari R Aflatoonian R Falak R Pourmand G Dehghani S Mortazavi M. Down-regulation of inflammatory signaling pathways despite up-regulation of toll-like receptors; the effects of corticosteroid therapy in brain-dead kidney donors, a double-blind, randomized, controlled trial. Mol Immunol (2018) 94:36–44. doi: 10.1016/j.molimm.2017.12.012 29253747

[74] Khan KN Kitajima M Fujishita A Nakashima M Masuzaki H. Toll-like receptor system and endometriosis. J Obstet Gynaecol Res (2013) 39(8):1281–92. doi: 10.1111/jog.12117 23855795

[75] Rehwinkel J Gack MU. Rig-I-Like receptors: Their regulation and roles in rna sensing. Nat Rev Immunol (2020) 20(9):537–51. doi: 10.1038/s41577-020-0288-3 PMC7094958 32203325

[76] Jiang H Swacha P Gekara NO. Nuclear Aim2-like receptors drive genotoxic tissue injury by inhibiting DNA repair. Adv Sci (Weinh) (2021) 8(22):e2102534. doi: 10.1002/advs.202102534 34658166 PMC8596118

[77] Gholamnezhadjafari R Tajik N Falak R Aflatoonian R Dehghan S Rezaei A. Innate inflammatory gene expression profiling in potential brain-dead donors: Detailed investigation of the effect of common corticosteroid therapy. Innate Immun (2017) 23(5):440–8. doi: 10.1177/1753425917709508 28504607

[78] Vercammen E Staal J Beyaert R. Sensing of viral infection and activation of innate immunity by toll-like receptor 3. Clin Microbiol Rev (2008) 21(1):13–25. doi: 10.1128/CMR.00022-07 18202435 PMC2223843

[79] Lesmeister MJ Jorgenson RL Young SL Misfeldt ML. 17beta-estradiol suppresses Tlr3-induced cytokine and chemokine production in endometrial epithelial cells. Reprod Biol Endocrinol (2005) 3(1):74. doi: 10.1186/1477-7827-3-74 16384532 PMC1343560

[80] Jiang C Liu C Guo J Chen L Luo N Qu X. The expression of toll-like receptors in eutopic and ectopic endometrium and its implication in the inflammatory pathogenesis of adenomyosis. Sci Rep (2017) 7(1):7365. doi: 10.1038/s41598-017-07859-5 28779087 PMC5544718

[81] Almasi MZ Hosseini E Jafari R Aflatoonian K Aghajanpour S Ramazanali F. Evaluation of toll-like receptor 3 (Tlr3) signaling pathway genes and its genetic polymorphisms in ectopic and eutopic endometrium of women with endometriosis. J Gynecol Obstet Hum Reprod (2021) 50(9):102153. doi: 10.1016/j.jogoh.2021.102153 33892179

[82] Herington JL Bruner-Tran KL Lucas JA Osteen KG. Immune interactions in endometriosis. Expert Rev Clin Immunol (2011) 7(5):611–26. doi: 10.1586/eci.11.53 PMC3204940 21895474

[83] Shen KY Liu HY Yan WL Wu CC Lee MH Leng CH. Liposomal Tlr9 agonist combined with Tlr2 agonist-fused antigen can modulate tumor microenvironment through dendritic cells. Cancers (Basel) (2020) 12(4):810. doi: 10.3390/cancers12040810 32231003 PMC7225995

[84] Oliveira-Nascimento L Massari P Wetzler LM. The role of Tlr2 in infection and immunity. Front Immunol (2012) 3:79. doi: 10.3389/fimmu.2012.00079 22566960 PMC3342043

[85] Marino A Pergolizzi S Cimino F Lauriano ER Speciale A D’Angelo V. Role of herpes simplex envelope glycoprotein b and toll-like receptor 2 in ocular inflammation: An ex vivo organotypic rabbit corneal model. Viruses (2019) 11(9):819. doi: 10.3390/v11090819 31487910 PMC6783931

[86] Sobstyl M Niedzwiedzka-Rystwej P Grywalska E Korona-Glowniak I Sobstyl A Bednarek W. Toll-like receptor 2 expression as a new hallmark of advanced endometriosis. Cells (2020) 9(8):1813. doi: 10.3390/cells9081813 32751735 PMC7464841

[87] Fazeli A Bruce C Anumba DO. Characterization of toll-like receptors in the female reproductive tract in humans. Hum Reprod (2005) 20(5):1372–8. doi: 10.1093/humrep/deh775 15695310

[88] Schaefer TM Desouza K Fahey JV Beagley KW Wira CR. Toll-like receptor (Tlr) expression and tlr-mediate Cytokine/Chemokine production by human uterine epithelial cells. Immunology (2004) 112(3):428–36. doi: 10.1111/j.1365-2567.2004.01898.x PMC1782499 15196211

[89] Tone K Stappers MHT Willment JA Brown GD. C-type lectin receptors of the dectin-1 cluster: Physiological roles and involvement in disease. Eur J Immunol (2019) 49(12):2127–33. doi: 10.1002/eji.201847536 PMC6916577 31580478

[90] Geijtenbeek TB Gringhuis SI. C-type lectin receptors in the control of T helper cell differentiation. Nat Rev Immunol (2016) 16(7):433–48. doi: 10.1038/nri.2016.55 27291962

[91] Brown GD Willment JA Whitehead L. C-type lectins in immunity and homeostasis. Nat Rev Immunol (2018) 18(6):374–89. doi: 10.1038/s41577-018-0004-8 29581532

[92] Keumatio Doungstop BC van Vliet SJ van Ree R de Jong EC van Kooyk Y. Carbohydrates in allergy: From disease to novel immunotherapies. Trends Immunol (2021) 42(7):635–48. doi: 10.1016/j.it.2021.05.002 34052120

[93] Wei C Mei J Tang L Liu Y Li D Li M. 1-Methyl-Tryptophan attenuates regulatory T cells differentiation due to the inhibition of estrogen-Ido1-Mrc2 axis in endometriosis. Cell Death Dis (2016) 7(12):e2489. doi: 10.1038/cddis.2016.375 27906184 PMC5260991

[94] Izumi G Koga K Takamura M Makabe T Nagai M Urata Y. Mannose receptor is highly expressed by peritoneal dendritic cells in endometriosis. Fertil Steril (2017) 107(1):167–73 e2. doi: 10.1016/j.fertnstert.2016.09.036 27793384

[95] Xiaocui L Wei H Yunlang C Zhenzhen Z Min A. Csf-1-Induced dc-sign(+) macrophages are present in the ovarian endometriosis. Reprod Biol Endocrinol (2022) 20(1):48. doi: 10.1186/s12958-022-00901-w 35260161 PMC8903642

[96] Zou Y Zhou JY Wang F Zhang ZY Liu FY Luo Y. Analysis of Card10 and Card11 somatic mutations in patients with ovarian endometriosis. Oncol Lett (2018) 16(1):491–6. doi: 10.3892/ol.2018.8659 PMC6006461 29928437

[97] Manek R Pakzamir E Mhawech-Fauceglia P Pejovic T Sowter H Gayther SA. Targeting src in endometriosis-associated ovarian cancer. Oncogenesis (2016) 5(8):e251. doi: 10.1038/oncsis.2016.54 27526105 PMC5007828

[98] Meggyes M Szereday L Bohonyi N Koppan M Szegedi S Marics-Kutas A. Different expression pattern of Tim-3 and galectin-9 molecules by peripheral and peritoneal lymphocytes in women with and without endometriosis. Int J Mol Sci (2020) 21(7):2343. doi: 10.3390/ijms21072343 32231038 PMC7177301

[99] Haller-Kikkatalo K Sarapik A Faure GC Bene MC Massin F Salumets A. Serum strem-1 (Soluble triggering receptor expressed on myeloid cells-1) associates negatively with embryo quality in infertility patients. Am J Reprod Immunol (2012) 68(1):68–74. doi: 10.1111/j.1600-0897.2011.01102.x 22229451

[100] Ting JP Lovering RC Alnemri ES Bertin J Boss JM Davis BK. The nlr gene family: A standard nomenclature. Immunity (2008) 28(3):285–7. doi: 10.1016/j.immuni.2008.02.005 PMC2630772 18341998

[101] Platnich JM Muruve DA. Nod-like receptors and inflammasomes: A review of their canonical and non-canonical signaling pathways. Arch Biochem Biophys (2019) 670:4–14. doi: 10.1016/j.abb.2019.02.008 30772258

[102] Wilmanski JM Petnicki-Ocwieja T Kobayashi KS. Nlr proteins: Integral members of innate immunity and mediators of inflammatory diseases. J Leukoc Biol (2008) 83(1):13–30. doi: 10.1189/jlb.0607402 17875812 PMC3256237

[103] Grivennikov SI Greten FR Karin M. Immunity, inflammation, and cancer. Cell (2010) 140(6):883–99. doi: 10.1016/j.cell.2010.01.025 PMC2866629 20303878

[104] Wang JQ Liu YR Xia Q Chen RN Liang J Xia QR. Emerging roles for Nlrc5 in immune diseases. Front Pharmacol (2019) 10:1352. doi: 10.3389/fphar.2019.01352 31824312 PMC6880621

[105] Neerincx A Rodriguez GM Steimle V Kufer TA. Nlrc5 controls basal mhc class I gene expression in an mhc enhanceosome-dependent manner. J Immunol (2012) 188(10):4940–50. doi: 10.4049/jimmunol.1103136 22490867

[106] Kobayashi KS van den Elsen PJ. Nlrc5: A key regulator of mhc class I-dependent immune responses. Nat Rev Immunol (2012) 12(12):813–20. doi: 10.1038/nri3339 23175229

[107] Kuenzel S Till A Winkler M Hasler R Lipinski S Jung S. The nucleotide-binding oligomerization domain-like receptor Nlrc5 is involved in ifn-dependent antiviral immune responses. J Immunol (2010) 184(4):1990–2000. doi: 10.4049/jimmunol.0900557 20061403

[108] Cho SX Vijayan S Yoo JS Watanabe T Ouda R An N. Mhc class I transactivator Nlrc5 in host immunity, cancer and beyond. Immunology (2021) 162(3):252–61. doi: 10.1111/imm.13235 PMC7884647 32633419

[109] Wu Y Shi T Li J. Nlrc5: A paradigm for nlrs in immunological and inflammatory reaction. Cancer Lett (2019) 451:92–9. doi: 10.1016/j.canlet.2019.03.005 30867141

[110] Yoshihama S Vijayan S Sidiq T Kobayashi KS. Nlrc5/Cita: A key player in cancer immune surveillance. Trends Cancer (2017) 3(1):28–38. doi: 10.1016/j.trecan.2016.12.003 28718425 PMC5518632

[111] Shukla A Cloutier M Appiya Santharam M Ramanathan S Ilangumaran S. The mhc class-I transactivator Nlrc5: Implications to cancer immunology and potential applications to cancer immunotherapy. Int J Mol Sci (2021) 22(4):1964. doi: 10.3390/ijms22041964 33671123 PMC7922096

[112] Wang Y Huang C Bian E Lei T Lv X Li J. Nlrc5 negatively regulates inflammatory responses in lps-induced acute lung injury through nf-kappab and P38 mapk signal pathways. Toxicol Appl Pharmacol (2020) 403:115150. doi: 10.1016/j.taap.2020.115150 32710960

[113] Zhan L Yao S Sun S Su Q Li J Wei B. Nlrc5 and autophagy combined as possible predictors in patients with endometriosis. Fertil Steril (2018) 110(5):949–56. doi: 10.1016/j.fertnstert.2018.06.028 30316442

[114] He R Liu X Zhang J Wang Z Wang W Fu L. Nlrc5 inhibits inflammation of secretory phase ectopic endometrial stromal cells by up-regulating autophagy in ovarian endometriosis. Front Pharmacol (2020) 11:1281. doi: 10.3389/fphar.2020.01281 33013364 PMC7461939

[115] Kelley N Jeltema D Duan Y He Y. The Nlrp3 inflammasome: An overview of mechanisms of activation and regulation. Int J Mol Sci (2019) 20(13):3328. doi: 10.3390/ijms20133328 31284572 PMC6651423

[116] Kelly P Meade KG O'Farrelly C. Non-canonical inflammasome-mediated il-1beta production by primary endometrial epithelial and stromal fibroblast cells is Nlrp3 and caspase-4 dependent. Front Immunol (2019) 10:102. doi: 10.3389/fimmu.2019.00102 30804935 PMC6371858

[117] Elliott EI Sutterwala FS. Initiation and perpetuation of Nlrp3 inflammasome activation and assembly. Immunol Rev (2015) 265(1):35–52. doi: 10.1111/imr.12286 25879282 PMC4400874

[118] Huang Y Xu W Zhou R. Nlrp3 inflammasome activation and cell death. Cell Mol Immunol (2021) 18(9):2114–27. doi: 10.1038/s41423-021-00740-6 PMC8429580 34321623

[119] He W-t Wan H Hu L Chen P Wang X Huang Z. Gasdermin d is an executor of pyroptosis and required for interleukin-1 beta secretion. Cell Res (2015) 25(12):1285–98. doi: 10.1038/cr.2015.139 PMC4670995 26611636

[120] Jorgensen I Miao EA. Pyroptotic cell death defends against intracellular pathogens. Immunol Rev (2015) 265(1):130–42. doi: 10.1111/imr.12287 PMC4400865 25879289

[121] Mai W Xu Y Xu J Zhao D Ye L Yu G. Berberine inhibits nod-like receptor family pyrin domain containing 3 inflammasome activation and pyroptosis in nonalcoholic steatohepatitis *Via* the Ros/Txnip axis. Front Pharmacol (2020) 11:185. doi: 10.3389/fphar.2020.00185 32194416 PMC7063468

[122] Han SJ Jung SY Wu SP Hawkins SM Park MJ Kyo S. Estrogen receptor beta modulates apoptosis complexes and the inflammasome to drive the pathogenesis of endometriosis. Cell (2015) 163(4):960–74. doi: 10.1016/j.cell.2015.10.034 PMC4640214 26544941

[123] Zhao J Ma W Chen W Gao J Li C Tong Y. Aeg-1 aggravates inflammation *Via* promoting Nalp3 inflammasome formation in murine endometriosis lesions. Anim Cells Syst (Seoul) (2019) 23(6):407–13. doi: 10.1080/19768354.2019.1691052 PMC6913626 31853378

[124] Hang Y Tan L Chen Q Liu Q Jin Y. E3 ubiquitin ligase Trim24 deficiency promotes Nlrp3/Caspase-1/Il-1beta-Mediated pyroptosis in endometriosis. Cell Biol Int (2021) 45(7):1561–70. doi: 10.1002/cbin.11592 33724611

[125] Guo X Xu X Li T Yu Q Wang J Chen Y. Nlrp3 inflammasome activation of mast cells by estrogen *Via* the nuclear-initiated signaling pathway contributes to the development of endometriosis. Front Immunol (2021) 12:749979. doi: 10.3389/fimmu.2021.749979 34630429 PMC8494307

[126] Choi J Jo M Lee E Kim SE Lee DY Choi D. Inhibition of the Nlrp3 inflammasome by progesterone is attenuated by abnormal autophagy induction in endometriotic cyst stromal cells: Implications for endometriosis. Mol Hum Reprod (2022) 28(4):gaac007. doi: 10.1093/molehr/gaac007 35333355

[127] Murakami M Osuka S Muraoka A Hayashi S Bayasula Kasahara Y. Effectiveness of Nlrp3 inhibitor as a non-hormonal treatment for ovarian endometriosis. Reprod Biol Endocrinol (2022) 20(1):58. doi: 10.1186/s12958-022-00924-3 35351143 PMC8966161

[128] Fan X Jin T. Structures of rig-I-Like receptors and insights into viral rna sensing. Struct Immunol (2019) 1172:157–88. doi: 10.1007/978-981-13-9367-9_8 31628656

[129] Brisse M Ly H. Comparative structure and function analysis of the rig-I-Like receptors: Rig-I and Mda5. Front Immunol (2019) 10:1586. doi: 10.3389/fimmu.2019.01586 31379819 PMC6652118

[130] Wu B Hur S. How rig-I like receptors activate mavs. Curr Opin Virol (2015) 12:91–8. doi: 10.1016/j.coviro.2015.04.004 PMC4470786 25942693

[131] Luangsay S Gruffaz M Isorce N Testoni B Michelet M Faure-Dupuy S. Early inhibition of hepatocyte innate responses by hepatitis b virus. J Hepatol (2015) 63(6):1314–22. doi: 10.1016/j.jhep.2015.07.014 26216533

[132] Wicherska-Pawlowska K Wrobel T Rybka J. Toll-like receptors (Tlrs), nod-like receptors (Nlrs), and rig-I-Like receptors (Rlrs) in innate immunity. tlrs, nlrs, and rlrs ligands as immunotherapeutic agents for hematopoietic diseases. Int J Mol Sci (2021) 22(24):13397. doi: 10.3390/ijms222413397 34948194 PMC8704656

[133] Hu B Jin C Li H-B Tong J Ouyang X Cetinbas NM. The DNA-sensing Aim2 inflammasome controls radiation-induced cell death and tissue injury. Science (2016) 354(6313):765–8. doi: 10.1126/science.aaf7532 PMC5640175 27846608

[134] Lammert CR Frost EL Bellinger CE Bolte AC McKee CA Hurt ME. Aim2 inflammasome surveillance of DNA damage shapes neurodevelopment. Nature (2020) 580(7805):647–52. doi: 10.1038/s41586-020-2174-3 PMC7788527 32350463

[135] Brunette RL Young JM Whitley DG Brodsky IE Malik HS Stetson DB. Extensive evolutionary and functional diversity among mammalian Aim2-like receptors. J Exp Med (2012) 209(11):1969–83. doi: 10.1084/jem.20121960 PMC3478938 23045604

[136] Fernandes-Alnemri T Yu J-W Datta P Wu J Alnemri ES. Aim2 activates the inflammasome and cell death in response to cytoplasmic DNA. Nature (2009) 458(7237):509–13. doi: 10.1038/nature07710 PMC2862225 19158676

[137] Wang B Tian Y Yin Q. Aim2 inflammasome assembly and signaling. Adv Exp Med Biol (2019) 1172:143–55. doi: 10.1007/978-981-13-9367-9_7 31628655

[138] Smatlik N Drexler SK Burian M Rocken M Yazdi AS. Asc speck formation after inflammasome activation in primary human keratinocytes. Oxid Med Cell Longev (2021) 2021:7914829. doi: 10.1155/2021/7914829 34777694 PMC8589508

[139] Schnappauf O Chae JJ Kastner DL Aksentijevich I. The pyrin inflammasome in health and disease. Front Immunol (2019) 10:1745. doi: 10.3389/fimmu.2019.01745 31456795 PMC6698799

[140] Lupfer C Anand PK. Integrating inflammasome signaling in sexually transmitted infections. Trends Immunol (2016) 37(10):703–14. doi: 10.1016/j.it.2016.08.004 PMC5086000 27592079

[141] Zhu H Zhao M Chang C Chan V Lu Q Wu H. The complex role of Aim2 in autoimmune diseases and cancers. Immun Inflammation Dis (2021) 9(3):649–65. doi: 10.1002/iid3.443 PMC8342223 34014039

[142] Sharma M de Alba E. Structure, activation and regulation of Nlrp3 and Aim2 inflammasomes. Int J Mol Sci (2021) 22(2):872. doi: 10.3390/ijms22020872 33467177 PMC7830601

[143] Lugrin J Martinon F. The Aim2 inflammasome: Sensor of pathogens and cellular perturbations. Immunol Rev (2018) 281(1):99–114. doi: 10.1111/imr.12618 29247998

[144] Wallet P Benaoudia S Mosnier A Lagrange B Martin A Lindgren H. Ifn-gamma extends the immune functions of guanylate binding proteins to inflammasome-independent antibacterial activities during francisella novicida infection. PloS Pathog (2017) 13(10):e1006630. doi: 10.1371/journal.ppat.1006630 28968459 PMC5624647

[145] Kumari P Russo AJ Shivcharan S Rathinam VA. Aim2 in health and disease: Inflammasome and beyond. Immunol Rev (2020) 297(1):83–95. doi: 10.1111/imr.12903 32713036 PMC7668394

[146] Sharma BR Karki R Kanneganti TD. Role of Aim2 inflammasome in inflammatory diseases, cancer and infection. Eur J Immunol (2019) 49(11):1998–2011. doi: 10.1002/eji.201848070 31372985 PMC7015662

[147] Choubey D Panchanathan R. Ifi16, an amplifier of DNA-damage response: Role in cellular senescence and aging-associated inflammatory diseases. Ageing Res Rev (2016) 28:27–36. doi: 10.1016/j.arr.2016.04.002 27063514

[148] Ansari MA Dutta S Veettil MV Dutta D Iqbal J Kumar B. Herpesvirus genome recognition induced acetylation of nuclear Ifi16 is essential for its cytoplasmic translocation, inflammasome and ifn-beta responses. PloS Pathog (2015) 11(7):e1005019. doi: 10.1371/journal.ppat.1005019 26134128 PMC4489722

[149] Choubey D. DNA-Responsive inflammasomes and their regulators in autoimmunity. Clin Immunol (2012) 142(3):223–31. doi: 10.1016/j.clim.2011.12.007 PMC3288811 22245264

[150] Asefa B Klarmann KD Copeland NG Gilbert DJ Jenkins NA Keller JR. The interferon-inducible P200 family of proteins: A perspective on their roles in cell cycle regulation and differentiation. Blood Cells Mol Dis (2004) 32(1):155–67. doi: 10.1016/j.bcmd.2003.10.002 14757431

[151] Diner BA Li T Greco TM Crow MS Fuesler JA Wang J. The functional interactome of pyhin immune regulators reveals ifix is a sensor of viral DNA. Mol Syst Biol (2015) 11(1):787. doi: 10.15252/msb.20145808 25665578 PMC4358659

[152] Santulli P Marcellin L Tosti C Chouzenoux S Cerles O Borghese B. Map kinases and the inflammatory signaling cascade as targets for the treatment of endometriosis? Expert Opin Ther Targets (2015) 19(11):1465–83. doi: 10.1517/14728222.2015.1090974 26389657

[153] Falcone T Flyckt R. Clinical management of endometriosis. Obstet Gynecol (2018) 131(3):557–71. doi: 10.1097/AOG.0000000000002469 29420391

[154] Obstetricians ACo, Gynecologists. Management of endometriosis. acog practice bulletin no. 114. Obstetrics Gynecology (2010) 116(1):225–36. 10.1097/AOG.0b013e3181e8b073 20567196

[155] Practice Committee of the American Society for Reproductive M.. Treatment of pelvic pain associated with endometriosis: A committee opinion. Fertil Steril (2014) 101(4):927–35. doi: 10.1016/j.fertnstert.2014.02.012 24630080

[156] Becker CM Gattrell WT Gude K Singh SS. Reevaluating response and failure of medical treatment of endometriosis: A systematic review. Fertil Steril (2017) 108(1):125–36. doi: 10.1016/j.fertnstert.2017.05.004 PMC5494290 28668150

[157] de Ziegler D Pirtea P Carbonnel M Poulain M Cicinelli E Bulletti C. Assisted reproduction in endometriosis. Best Pract Res Clin Endocrinol Metab (2019) 33(1):47–59. doi: 10.1016/j.beem.2018.10.001 30503728

[158] Wang X Shen B Sun D Cui X. Aspirin ameliorates cerebral infarction through regulation of Tlr4/Nfkappabmediated endoplasmic reticulum stress in mouse model. Mol Med Rep (2018) 17(1):479–87. doi: 10.3892/mmr.2017.7879 29115440

[159] Zhang P Peng H Gao C Fan Z Xia Z. Aspirin-triggered lipoxin protects lipopolysaccharide-induced acute kidney injury *Via* the Tlr4/Myd88/Nf-κb pathway. Saudi J Kidney Dis Transplant (2020) 31(5):937. doi: 10.4103/1319-2442.301200 33229758

[160] Li F Xu D Hou K Gou X Lv N Fang W. Pretreatment of indobufen and aspirin and their combinations with clopidogrel or ticagrelor alleviates inflammasome mediated pyroptosis *Via* inhibiting nf-Kappab/Nlrp3 pathway in ischemic stroke. J Neuroimmune Pharmacol (2021) 16(4):835–53. doi: 10.1007/s11481-020-09978-9 33512659

[161] Tayel SS Helmy AA Ahmed R Esmat G Hamdi N Abdelaziz AI. Progesterone suppresses interferon signaling by repressing tlr-7 and mxa expression in peripheral blood mononuclear cells of patients infected with hepatitis c virus. Arch Virol (2013) 158(8):1755–64. doi: 10.1007/s00705-013-1673-z 23525700

[162] Tang H Hua F Wang J Yousuf S Atif F Sayeed I. Progesterone and vitamin d combination therapy modulates inflammatory response after traumatic brain injury. Brain Inj (2015) 29(10):1165–74. doi: 10.3109/02699052.2015.1035330 PMC4894830 26083048

[163] Hong Y Liu Y Yu D Wang M Hou Y. The neuroprotection of progesterone against abeta-induced Nlrp3-Caspase-1 inflammasome activation *Via* enhancing autophagy in astrocytes. Int Immunopharmacol (2019) 74:105669. doi: 10.1016/j.intimp.2019.05.054 31176046

[164] Espinosa-Garcia C Atif F Yousuf S Sayeed I Neigh GN Stein DG. Progesterone attenuates stress-induced Nlrp3 inflammasome activation and enhances autophagy following ischemic brain injury. Int J Mol Sci (2020) 21(11):3740. doi: 10.3390/ijms21113740 32466385 PMC7312827

[165] Matias ML Romao-Veiga M Ribeiro VR Nunes PR Gomes VJ Devides AC. Progesterone and vitamin d downregulate the activation of the Nlrp1/Nlrp3 inflammasomes and Tlr4-Myd88-Nf-Kappab pathway in monocytes from pregnant women with preeclampsia. J Reprod Immunol (2021) 144:103286. doi: 10.1016/j.jri.2021.103286 33578174

[166] Sobesky JL D'Angelo HM Weber MD Anderson ND Frank MG Watkins LR. Glucocorticoids mediate short-term high-fat diet induction of neuroinflammatory priming, the Nlrp3 inflammasome, and the danger signal Hmgb1. eNeuro (2016) 3(4):ENEURO.0113–16. doi: 10.1523/ENEURO.0113-16.2016 PMC5004086 27595136

[167] Zhang B Zhang Y Xu T Yin Y Huang R Wang Y. Chronic dexamethasone treatment results in hippocampal neurons injury due to activate Nlrp1 inflammasome in vitro. Int Immunopharmacol (2017) 49:222–30. doi: 10.1016/j.intimp.2017.05.039 28605710

[168] Srivastava MD Thomas A Srivastava BI Check JH. Expression and modulation of progesterone induced blocking factor (Pibf) and innate immune factors in human leukemia cell lines by progesterone and mifepristone. Leuk Lymphoma (2007) 48(8):1610–7. doi: 10.1080/10428190701471999 17701593

[169] Mita S Shimizu Y Notsu T Imada K Kyo S. Dienogest inhibits toll-like receptor 4 expression induced by costimulation of lipopolysaccharide and high-mobility group box 1 in endometrial epithelial cells. Fertil Steril (2011) 96(6):1485–9 e4. doi: 10.1016/j.fertnstert.2011.09.040 22014880

[170] Iwanaszko M Kimmel M. Nf-κb and irf pathways: Cross-regulation on target genes promoter level. BMC Genomics (2015) 16(1):1–8. doi: 10.1186/s12864-015-1511-7 25888367 PMC4430024

[171] Gruber TM Mechsner S. Pathogenesis of endometriosis: The origin of pain and subfertility. Cells (2021) 10(6):1381. doi: 10.3390/cells10061381 34205040 PMC8226491

[172] Thakur KK Saini J Mahajan K Singh D Jayswal DP Mishra S. Therapeutic implications of toll-like receptors in peripheral neuropathic pain. Pharmacol Res (2017) 115:224–32. doi: 10.1016/j.phrs.2016.11.019 27894923

[173] Bruno K Woller SA Miller YI Yaksh TL Wallace M Beaton G. Targeting toll-like receptor-4 (Tlr4)-an emerging therapeutic target for persistent pain states. Pain (2018) 159(10):1908–15. doi: 10.1097/j.pain.0000000000001306 PMC7890571 29889119

[174] Elzinga S Murdock BJ Guo K Hayes JM Tabbey MA Hur J. Toll-like receptors and inflammation in metabolic neuropathy; a role in early versus late disease? Exp Neurol (2019) 320:112967. doi: 10.1016/j.expneurol.2019.112967 31145897 PMC6708507

[175] Chiu KM Lu CW Lee MY Wang MJ Lin TY Wang SJ. Neuroprotective and anti-inflammatory effects of lidocaine in kainic acid-injected rats. Neuroreport (2016) 27(7):501–7. doi: 10.1097/WNR.0000000000000570 26999361

[176] Hermanns H Hollmann MW Stevens MF Lirk P Brandenburger T Piegeler T. Molecular mechanisms of action of systemic lidocaine in acute and chronic pain: A narrative review. Br J Anaesth (2019) 123(3):335–49. doi: 10.1016/j.bja.2019.06.014 31303268

[177] Wickstrom K Bruse C Sjosten A Spira J Edelstam G. Pertubation with lignocaine as a new treatment of dysmenorrhea due to endometriosis: A randomized controlled trial. Hum Reprod (2012) 27(3):695–701. doi: 10.1093/humrep/der434 22232129

[178] Lee PY Tsai PS Huang YH Huang CJ. Inhibition of toll-like receptor-4, nuclear factor-kappab and mitogen-activated protein kinase by lignocaine may involve voltage-sensitive sodium channels. Clin Exp Pharmacol Physiol (2008) 35(9):1052–8. doi: 10.1111/j.1440-1681.2008.04962.x 18505446

[179] Wang L Wang M Li S Wu H Shen Q Zhang S. Nebulized lidocaine ameliorates allergic airway inflammation *Via* downregulation of Tlr2. Mol Immunol (2018) 97:94–100. doi: 10.1016/j.molimm.2018.03.010 29609129

